# Subjective, behavioural and physiological correlates of stress in women using hormonal contraceptives

**DOI:** 10.1192/bjp.2025.7

**Published:** 2025-06

**Authors:** Zoé Bürger, Charlotte Kordowich, Julia Kübbeler, Carolin Müllerschön, Ann-Christin S. Kimmig, Min Su, Michael Lämmerhofer, Julia Sacher, Melanie Henes, Erika Comasco, Birgit Derntl, Lydia Kogler

**Affiliations:** Department of Psychiatry and Psychotherapy, Tübingen Center for Mental Health (TüCMH), University of Tübingen, Tübingen, Germany; Department of Women’s and Children’s Health, Science for Life Laboratory, Uppsala University, Uppsala, Sweden; Department of Pharmacy and Biochemistry, Institute of Pharmaceutical Sciences, Pharmaceutical (Bio-)Analysis, University of Tübingen, Tübingen, Germany; Cognitive Neuroendocrinology, Max Planck Institute for Human Cognitive and Brain Sciences, Leipzig, Germany; Clinic of Cognitive Neurology, University of Leipzig, Leipzig, Germany; Center for Integrated Female Health and Gender Medicine, Medical Faculty, University of Leipzig, Leipzig, Germany; Medical Department III – Endocrinology, Nephrology, Rheumatology, University of Leipzig, Leipzig, Germany; Department of Women’s Health, University of Tübingen, Tübingen, Germany; LEAD Graduate School & Research Network, University of Tübingen, Tübingen, Germany; German Center for Mental Health (DZPG), Partner Site Tübingen, Tübingen, Germany

**Keywords:** Stress, hormones, intrauterine device, hormonal contraception, menstrual cycle

## Abstract

**Background:**

Stress, a major risk factor for mental health problems, is influenced by hormonal fluctuations from the menstrual cycle and hormonal oral contraceptives (OC). Despite widespread use, the impact of hormonal intrauterine devices (IUDs) on stress is limited to one study.

**Aims:**

This study examines psychoendocrine stress responses in women using IUDs, OCs and women with a natural, regular menstrual cycle (NC) to better understand how endogenous and exogenous hormones influence stress.

**Method:**

Using a repeated-measures design, we investigated stress responses in IUD and OC users and NC women. The Maastricht Acute Stress Task and its control task were applied twice within 4 months to assess subjective, endocrine and physiological stress correlates. Detailed endogenous and exogenous hormonal profiles were obtained, and women completed a 7-day diary (via ecological momentary assessment) after each appointment.

**Results:**

Based on subjective, physiological and cortisol responses, stress induction was successful in all groups. IUD users reported higher subjective stress, negative affect and anxiety and lower positive affect compared to NC women. OC users exhibited a blunted cortisol response and higher heart rate but reported less acute stress and negative emotions than the other groups in the 7-day diary. Oestradiol and progesterone were suppressed in OC and IUD users compared with NC women. Progesterone, testosterone and oestradiol were differently associated with skin conductance, socio-emotional stress and negative affect.

**Conclusions:**

IUD and OC use distinctly affect stress response, possibly because of their diverging metabolic pathways and hormone levels. IUD users showed higher emotional reactivity to stress in both lab and daily life, while OCs influenced physiological correlates. These findings highlight that exogenous hormone administration, previously thought to have limited systemic effects, affects women’s psychological well-being, underscoring the need for further research into stress-related disorders among women using hormonal contraceptives.

Facing stressful situations is a natural part of life, and physical and emotional responses to these stressors shape the overall stress response. Sex and gender differences have been reported for stress responses, with sex hormones potentially playing a significant role.^1^ Two important pathways for the stress response are the hypothalamic–pituitary–adrenal (HPA) axis and the autonomic nervous system (ANS). While the latter is a rapid response system, upregulating bodily activity to prepare for potential threats (e.g. through increased cardiac output^2^), the HPA axis reacts more slowly, releasing cortisol, which typically peaks within 20–30 minutes after stress onset.^3^ The hypothalamic–pituitary–gonadal (HPG) axis, responsible for regulating sex hormones, is closely connected to the HPA axis and can influence its activity.^1^ Not only do cortisol responses vary throughout the menstrual cycle,^4^ but women using (combined) oral contraceptives (OCs) show a blunted cortisol response compared to natural, regular menstrual cycle (NC) women in the luteal phase, according to a meta-analysis of 14 studies involving over 1000 participants.^5^ Despite the relevance of stress for women’s mental health, research on stress, hormonal transitions and hormonal contraception remains scarce, especially concerning methods other than OCs, such as hormonal intrauterine devices (IUDs). Although IUDs are used by millions of women worldwide,^6^ only one study has investigated stress responses in women using IUDs so far. Contrary to the blunted cortisol in women using OCs, women using IUDs showed a highly potentiated cortisol response to acute stress and a heightened heart rate.^7^ Altered stress processing, as observed in IUD users, could affect several physiological and psychological outcomes including emotion regulation and mood.^1^ While women with IUDs show altered emotion regulation processes,^8^ findings on mood effects have been inconsistent (as shown in two reviews^9,10^). Recent research suggests an increased risk for depression and a higher likelihood of antidepressant prescription among IUD users,^11^ particularly at higher hormone doses.^12^

Importantly, the differences in stress responses between hormonal contraception methods may stem from variations in hormone composition and metabolic pathways. Combined OCs contain both ethinyl oestradiol (EE) and a progestin, while IUDs contain only the progestin levonorgestrel (LNG). OCs undergo hepatic first-pass metabolism, whereas LNG from IUDs directly enters the bloodstream through the uterine lining. This difference allows IUDs to achieve similar (contraceptive) efficacy with a lower hormone dose than OCs. However, LNG levels are rarely assessed in women using IUDs and OCs, and the interactions between hormone concentrations, both endogenous and exogenous, and stress responses, remain largely unexplored, despite the great need to better understand women’s increased vulnerability to stress-related disorders such as depression and anxiety. Here the assessment of endogenous and exogenous hormones may provide insight into mechanistic actions. Further, investigating how variations in hormonal composition and delivery methods influence different dimensions of the stress response (i.e. stress correlates) is needed.

In the present study, we therefore compared subjective, endocrine and physiological stress responses among women with different hormonal status: women using IUDs, women using OCs and NC women in the luteal phase. An acute psychosocial stress induction paradigm was implemented within a longitudinal design to assess intra-individual variability in stress responses over several months. Following stress induction, we expected (a) a potentiated cortisol response in women using IUDs and a blunted response in women using OCs,^7^ and (b) a higher heart rate in women using IUDs.^7^ Regarding group differences for subjective stress (including the 7-day diary) and skin conductance, as well as the direct impact of exogenous hormone concentrations, we had no directed *a priori* hypotheses because of lacking and inconsistent data in the existing literature.

## Method

### Participants

Healthy premenopausal women aged 18–40 years were recruited using flyers in gynaecological practices and email services of the University of Tübingen. We recruited three groups of women: (a) women using the LNG-IUD (IUD, *n* = 27), (b) women using OCs (*n* = 30) and (c) NC women (*n* = 29). Based on *a priori* power analyses (three groups, two measurement timepoints, error rate of 0.5, power of 0.95) and accounting for participants possibly dropping out, we aimed to recruit 25 women/group completing both timepoints.^13^ The women used their respective method for birth control for at least 6 months; details on the hormonal contraception are found in Supplement Table S1 available at https://doi.org/10.1192/bjp.2025.7. As all participants in our study were of female sex and identified as cisgender, we use the term ‘women’ throughout this paper when referring to our sample.^14,15^ A detailed description of inclusion and exclusion criteria can be found in the supplement. All women gave written informed consent. The authors assert that all procedures contributing to this work comply with the ethical standards of the relevant national and institutional committees on human experimentation and with the Helsinki Declaration of 1975, as revised in 2013. All procedures involving human participants were approved by the University Hospital of Tübingen (approval 067/2020).

### Study design

**Fig. 1:**
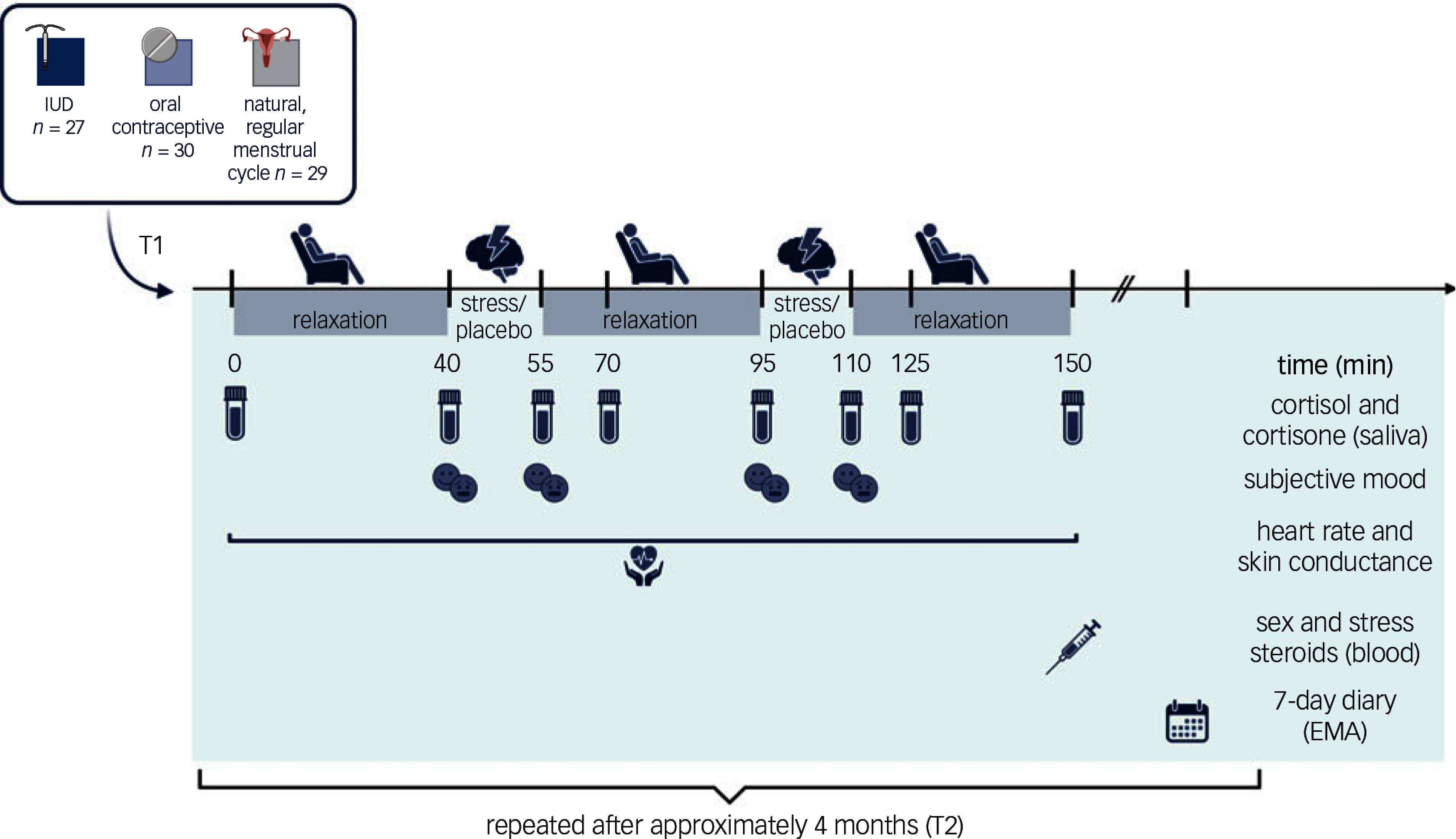
Detailed study design of one measurement day (T1), repeated after approximately 4 months (T2). Participants had to forgo taking medication in the 24 h prior to the appointment, refrain from consuming caffeine or exercising 3 h before, and from food and drinks, except water, for 1 h prior to testing. All appointments were scheduled on weekdays, starting between 13.00 h and 14.00 h. In the first 30 min after arrival, participants filled out self-report questionnaires. After applying electrodes for skin conductance and pulse-oximeter for heart rate measures, participants gave the first saliva sample, then underwent a 40 min relaxation phase watching non-arousing documentaries. A second saliva sample was given with a short subjective mood questionnaire. In a randomised fashion, either the stress or placebo task of the MAST was applied, followed by another saliva sample and subjective mood questionnaire. During a second 40 min relaxation phase, saliva was collected 15 min after the previous sample. Participants underwent the second part of the MAST (stress or placebo), and before and after, a saliva sample and a subjective mood questionnaire was collected. Participants watched a final 40 min relaxing documentary, and saliva samples were collected at 15 min and 40 min post-task. For 7 days, a daily mood and stress diary was filled out (7-day diary via ecological momentary assessment (EMA)). The second measurement day had the same timeline, was counter-balanced for task order (Supplement Table S2 for details) and was scheduled on average 4 months after the first time point. Created with BioRender.com. IUD, intrauterine device.

After a screening session to check inclusion and exclusion criteria, participants came in twice within 4 months (121 ± 37.4 days, timepoints T1 and T2) to assess intra-individual variability. They followed a strict protocol 24 h before each appointment to control influences on stress response, including refraining from caffeine consumption, exercise, etc. (Fig. 1 provides a description). Appointments were scheduled on weekday afternoons to account for diurnal cortisol fluctuations. Participants underwent the Maastricht Acute Stress Task (MAST^2^) and its non-stressful control (placebo) in a counterbalanced order (Supplement Table S6), with relaxation periods in between (for details on the MAST, see supplement). Cortisol/cortisone, subjective mood, heart rate, skin conductance and steroid hormones in blood were measured at specific times during the sessions (Fig. 1). The second timepoint T2 was identical to the first, with the exception that the task presentation order was reversed.

### General self-report questionnaires

Demographic information and questionnaire data assessing depressive symptoms (Beck Depression Inventory-II (BDI-II)^16^), trait anxiety (State-Trait Anxiety Inventory - Trait version (STAI-T)^17^), childhood trauma (Childhood Trauma Questionnaire (CTQ)^18^) and life stressors (Life Stressor Checklist-Revised (LSC-R)^19^), overall quality of life (World Health Organisation Quality of Life (WHOQOL)^20^) and stress- and hormonal status-related questionnaires were collected to assess and control for between-group inhomogeneity and exclude influences other than hormonal contraception use (see supplement).

### Task-related subjective stress and affect

Before and after each task, participants completed self-report questions (Presentation® Version 23.0, used in Windows; Neurobehavioral Systems, Inc., Berkeley, CA, USA; www.neurobs.com) assessing subjective stress (visual-analogue scale: from *not at all stressed* to *extremely stressed*), affect (Positive and Negative Affect Schedule (PANAS)^21^), state anxiety (State-Trait Anxiety Inventory - State version (STAI-S)^17^) and emotions (emotional self rating (ESR)^22^) (see supplement).

### Steroid hormones in plasma and salivary cortisol/cortisone

Plasma obtained from blood drawn at the end of each measurement day was analysed at the Institute of Pharmaceutical Sciences, University of Tübingen, Germany. Liquid chromatography-tandem mass spectrometry (LC-MS/MS) was used to determine hormone levels of testosterone, progesterone, oestradiol and cortisol, as well as EE and LNG, among others (see supplement). Saliva samples were collected using a synthetic fibre swab (Salivette^®^ Cortisol, Sarstedt, Nümbrecht, Germany) and analysed at the Institute of Pharmaceutical Sciences, University of Tübingen. Salivary cortisol and cortisone were determined by a micro-flow liquid chromatography-mass spectrometry assay.^23^

Detailed analysis procedures for plasma and saliva are described in the supplement.

### Physiological stress

Heart rate and skin conductance were recorded using a BIOPAC MP160 (Biopac Systems, Inc., Goleta, CA, USA). For detailed recording and processing methods, see the supplement.

### Seven-day diary (ecological momentary assessment)

After both measurement days, participants filled in a 7-day diary each evening, starting the day after the measurement, containing questionnaires pertaining to their well-being (PANAS,^21^ STAI-T,^17^ ESR^22^) and various stressors they might have encountered each day (Supplement Table S7).

### Statistical analysis

Data analysis, visualisation and statistics were done using RStudio (version 2023.9.1.494, used in Windows; Posit Software, PBC, Boston, MA, USA; R version 4.3.1); the packages are described in the supplement.

For the sample description, univariate analyses of variance (ANOVAs) with the between-subject factor *group* (IUD/OC/NC) were performed. All other variables were analysed using linear mixed models (LMMs) with a random intercept for each subject. Assumptions were checked and outliers identified via Cook’s distance; models excluding outliers are reported. When the main predictor *group* was included, models were run twice, with different reference groups: NC or IUD. The second model with IUD as the reference group was used to ascertain effects within hormonal contraception (i.e. IUD versus OC) and only effects pertaining to this difference were extracted from the second model.

#### Steroid hormones in plasma

Plasma levels of steroid hormones were measured in picogram per millilitre and converted to nanomole per litre (nmol/L), then log-transformed for statistical analysis, except for oestradiol, which was approximately normally distributed. Hormones were analysed with LMMs using the between-subjects factor *group* and the within-subject factor *timepoint* (T1/T2).

#### Salivary cortisol and cortisone

For analysis, cortisol and cortisone levels were transformed from ng/mL to nmol/L and the area under the curve with regard to increase (AUCi,^24^ reference: second saliva sample; three timepoints of 15, 30 and 55 min after task onset) was calculated for each participant and each task separately. In addition, the cortisol-to-cortisone ratio was calculated. LMMs with the between-subjects factor *group* and the within-subject factors *task* (placebo/MAST) and *timepoint* (T1/T2) and the interaction *group-by-task* were fitted.

#### Task-related subjective stress and affect

Ratings were measured before and after both the MAST and placebo task and the pre-values were added as continuous within-subject factor to account for baseline variations, with the post-values as dependent variable. The between-subjects factor *group* and the within-subject factors *task* (placebo/MAST) and *timepoint* (T1/T2) and the interaction *group-by-task* were added.

#### Physiological stress

Mean heart rate during stress and placebo tasks, the frequency of skin conductance responses (SCRs), tonic mean values and a global mean representing both phasic and tonic components of skin conductance were used as dependent variables, with the between-subjects factor *group* and the within-subject factors *task* (placebo/MAST) and *timepoint* and the interaction *group-by-task*.

#### Seven-day diary

The area under the curve (AUC)^24^ over the 7 days was computed for each question and used as a dependent variable in the LMMs. The between-subjects factor *group* and the within-subject factor *timepoint* (T1/T2) were used.

#### Exploratory hormone associations with stress correlates

Additional exploratory analyses were performed to assess whether sex hormones were predictive of stress correlates or 7-day diary-variables. We ran LMMs for all stress correlates, using only the stress and not placebo data, and significant 7-day diary variables, separated by group and sex hormones (as they were highly correlated). Models for oestradiol, progesterone and testosterone were run in all three groups, and additionally LNG models in IUD and OC users, and EE models in OC users. *Timepoint* was added as an additional factor but regarded as a covariate-of-no-interest in these analyses, as timepoint effects are already reported. To account for multiple testing in these exploratory analyses, false-discovery-rate (FDR) correction was applied within each group for each stress correlate separately (e.g. correction in women using IUDs for cortisol AUCi).

## Results

### Study population

**Fig. 2:**
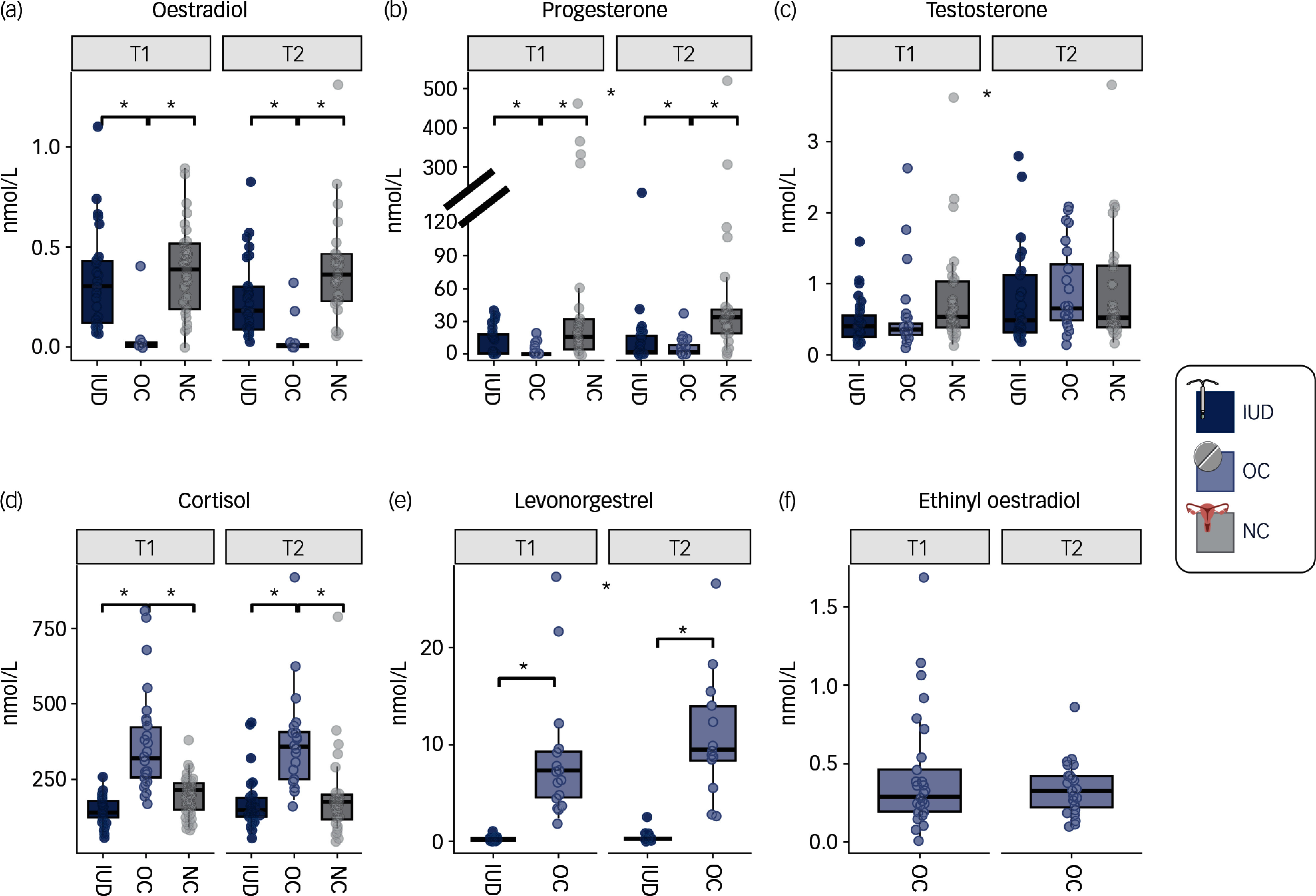
Endogenous and exogenous hormone levels in plasma across IUD users (dark blue), OC users (light blue) and NC women (grey) for both measurement timepoints (T1: first measurement; T2: ca. 4 months later; only task presentation order was switched). Sample sizes for T1 (IUD/OC/NC): Oestradiol: 27/13/29; Progesterone, Testosterone, Cortisol: 27/30/29; Levonorgestrel: 27/17/0; Ethinyl Oestradiol: 0/29/0. * indicates significance at *P* = 0.05. Created with BioRender.com. nmol/L, nanomole per litre; IUD, intrauterine device; OC, oral contraceptive; NC, natural, regular menstrual cycle.

Groups were comparable in age, depression score and trait anxiety at screening and chronic and perceived stress as well as further measures at timepoint T1 (see Supplement Table S2). However, life stressors weighted by affect within the past year were higher in women using IUDs than NC women (post hoc: *t*(83) = 2.59, *p*_*bonferroni*_ = 0.034, η^2^ = 0.08), although the number of life stressors experienced did not differ between groups (*p* = 0.420). To better characterise the groups, we compared baseline endogenous and exogenous sex hormone and cortisol levels in blood. For oestradiol and progesterone, NC women had the highest plasma concentrations, followed by IUD users, with OC users having the lowest concentrations (all *p*s < 0.026; Fig. 2; Supplement Table S3 for full statistics). Plasma cortisol showed the reverse pattern, with OC users having higher levels of cortisol compared with the other two groups (all *p*s < 0.001). Testosterone levels did not differ between the groups (*p* > 0.0662). LNG was significantly higher in OC users compared with IUD users (*p* < 0.001). Further, timepoint effects could be seen for testosterone, progesterone and LNG, with higher values at the second timepoint compared with the first (all *p*s < 0.002). Hormonal levels in our sample were consistent with those reported in previous studies examining hormonal levels in OC users and NC women^25,26^ and with normative ranges (Supplement Table S4).

### Successful stress induction

**Fig. 3:**
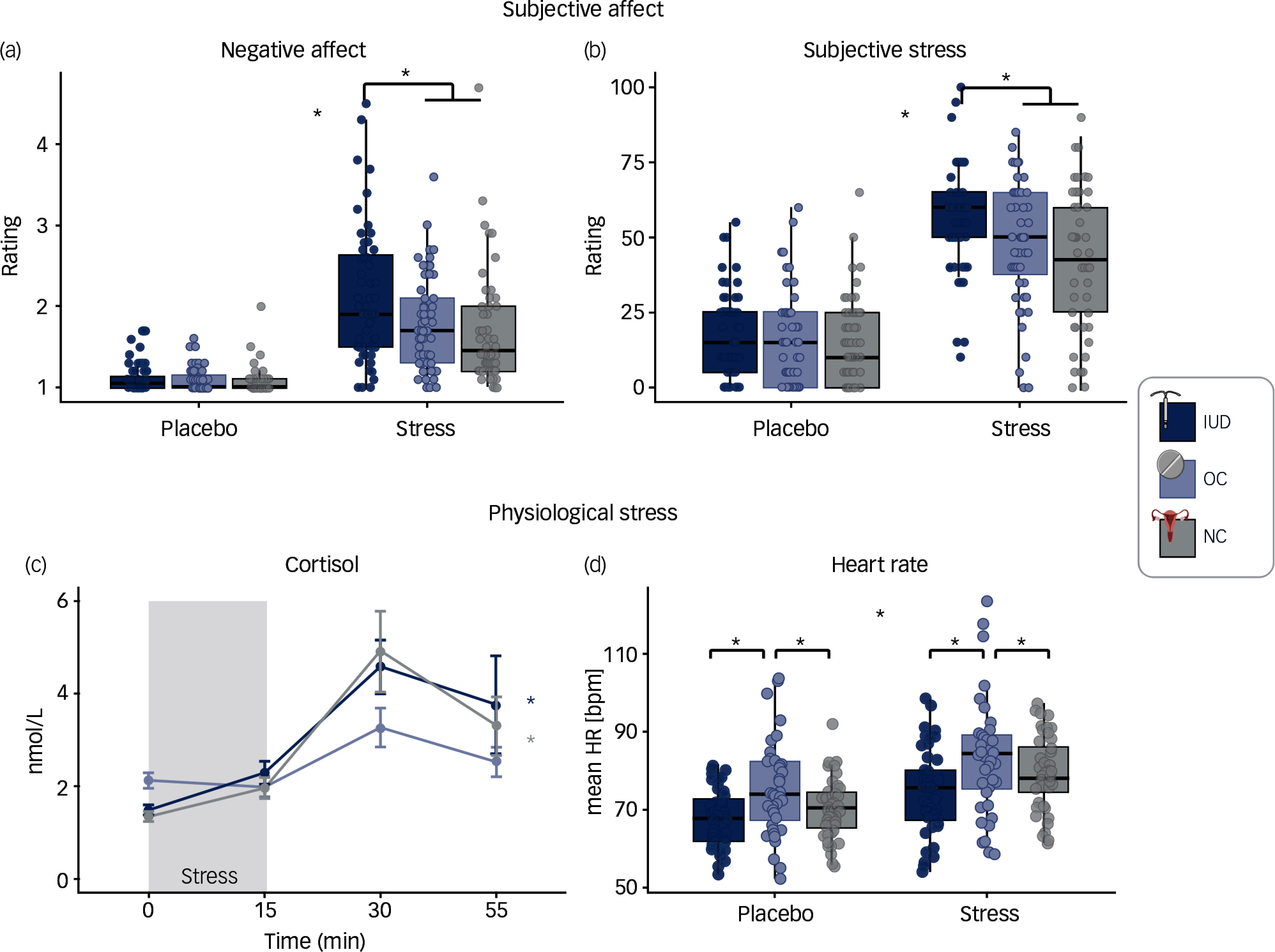
Stress correlates across IUD users (dark blue), OC users (light blue) and NC women (grey) for both measurement timepoints (T1 first measurement; T2 four months later). Top: subjective affect after placebo versus stress. (a) negative affect and (b) subjective stress. Bottom: physiological stress correlates. (c) cortisol response during stress task, stress onset at time 0, dots and bars indicate mean and s.e. (d) mean heart rate (HR) during placebo versus stress. * indicates significance at *P* = 0.05. Created with BioRender.com. IUD, intrauterine device; OC, oral contraceptive; NC, natural, regular menstrual cycle; nmol/L, nanomole per litre; HR, heart rate.

Stress induction was successful across multiple correlates of stress. Across all groups, negative affect, subjective stress and anxiety were higher after stress than after placebo (respectively: *b* = 0.57; *b* = 40.07; *b* = 12.12; all *p*s < 0.001; Figs. 3(a) and 3(b)). Cortisol response showed increased AUCi for stress, indicating higher cortisol values after stress induction compared with placebo (*b* = 103.23, *p* < 0.001). Physiologically, mean heart rate and all skin conductance measures increased during stress compared with placebo (mean heart rate: *b* = 6.27; SCR frequency: *b* = 5.18; tonic: *b* = 1.79; global: *b* = 1.98; all *p*s < 0.001).

### Repeated measures matter for the ANS but not cortisol or subjective stress

We assessed stress correlates twice across all women to assess intra-individual variability in stress responses over several months. From the first to the second timepoint (T1 to T2), only the task presentation order was reversed (Fig. 1). We found that repeated measures did not change subjective nor cortisol stress response (all *p*s > 0.101). Physiological stress response was affected by timepoint in all groups, as mean heart rate decreased from T1 to T2 (*b* = −2.55, *p* < 0.001; 95%CI and *t*-values in supplement). A similar pattern emerged for skin conductance, with lower frequency of SCRs and lower tonic and global skin conductance at the second timepoint compared with the first (respectively: *b* = −1.53, *b* = −1.88, *b* = −1.94; all *p*s < 0.001).

### Subjective stress altered in IUD users versus cortisol in OC users

Following stress induction, IUD users reported lower positive affect and higher negative affect, subjective stress and anxiety than NC women (respectively: *b* = −0.37, *p* = 0.008; *b* = 0.43, *p* < 0.001; *b* = 13.28, *p* = 0.003; *b* = 7.40, *p* = 0.003; Figs. 3(a) and 3(b)). In addition, negative affect was higher in IUD users than in OC users (*b* = −0.31, *p* = 0.009). IUD users did not differ from OC users for positive affect, subjective stress or anxiety (all *p*s > 0.758).

OC users compared with both other groups showed a blunted salivary cortisol response, with lower AUCi in the stress task (NC versus OC: *b* = −80.14, *p* = 0.007; OC versus IUD: *b* = −62.54; *p* = 0.033, Fig. 3(c), supplement Fig. S1). IUD users did not differ from NC women in their cortisol AUCi (*p* > 0.412). Cortisone AUCi and the cortisone-to-cortisol-ratio AUCi revealed the same pattern as cortisol AUCi (Supplement Figs. S2 and S3). Further, OC users exhibited a higher mean heart rate compared to NC women during both stress and placebo tasks (*b* = 6.52, *p* = 0.018). No group differences were found for any of the skin conductance measures (all *p*s > 0.113).

### Seven-day diary shows lower negative affect and stress in OC users

In the week following the lab visits, OC users reported lower levels of negative affect, negative emotions, acute work stress, anger and sadness than IUD users (all *p*s < 0.023; see Supplement Table S5 for full statistics) and fewer negative emotions and less acute socio-emotional stress than NC women (all *p*s < 0.023). No other assessed 7-day diary variable emerged as significant.

### Sex hormones affect stress response

**Fig. 4:**
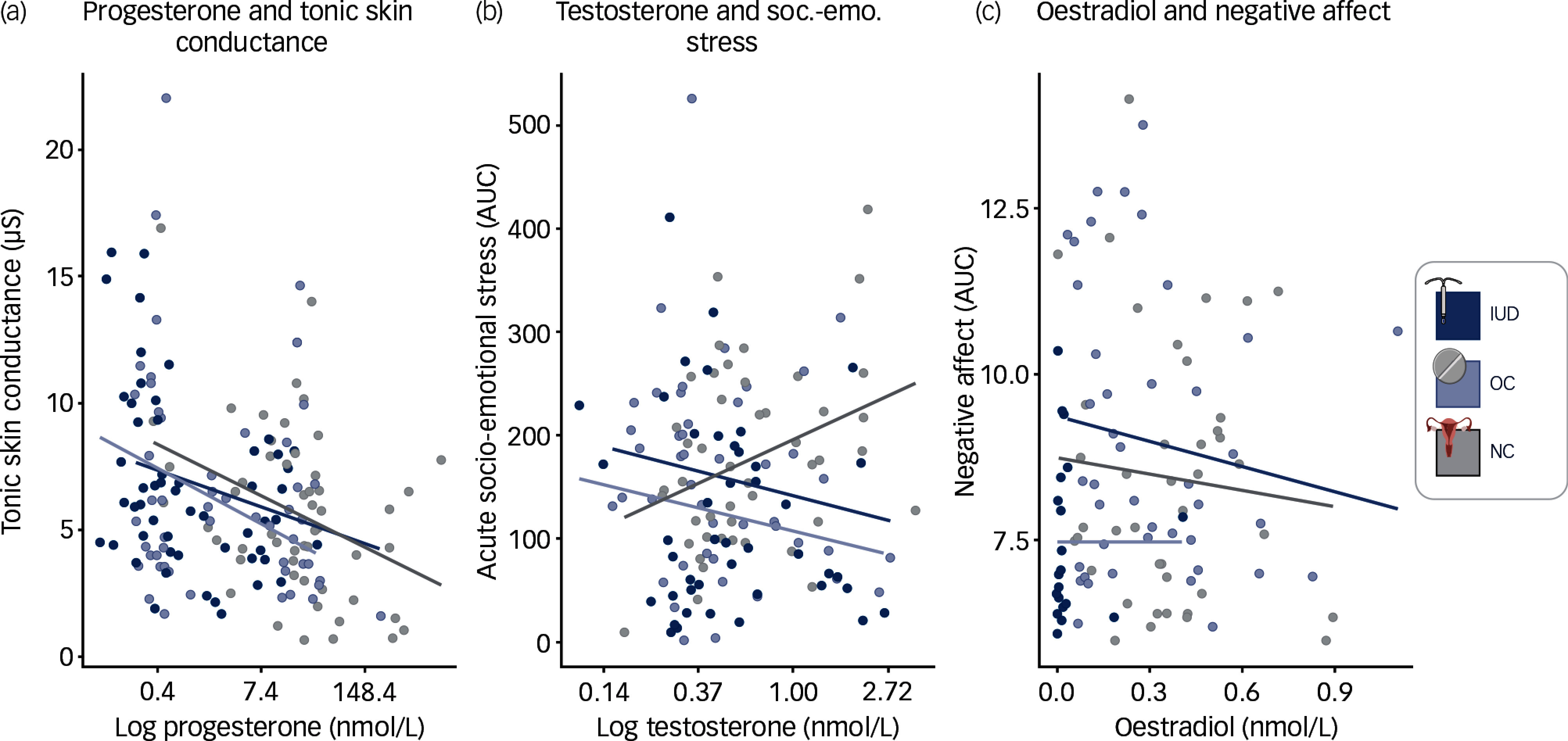
Sex hormone associations with stress correlates and 7-day diary across IUD users (dark blue), OC users (light blue) and NC women (grey) for both measurement timepoints (T1 first measurement; T2 four months later). (a) progesterone predicting tonic skin conductance, significant only for NC women but not IUD users or OC users. (b) testosterone positively predicts acute socio-emotional (soc.-emo) stress in 7-day diary among NC women but not IUD users or OC users. (c) oestradiol negatively predicts negative affect in 7-day diary among IUD users, but not OC users or NC women. Created with BioRender.com. soc.-emo., socio-emotional; IUD, intrauterine device; OC, oral contraceptive; NC, natural, regular menstrual cycle; AUC, area under the curve.

We conducted exploratory analyses to investigate whether levels of sex hormones at the two timepoints could predict stress correlates and 7-day diary outcomes. Across all groups, progesterone negatively predicted tonic skin conductance and global mean skin conductance, although this was only significant in NC women (respectively: *b* = −0.52, *p*_*FDR*_ values = 0.031; *b* = −0.54, *p*_*FDR*_ values = 0.034, Fig. 4(a), see supplement for full statistics). For the 7-day diary variables that showed significant group differences, associations with sex hormones emerged. In NC women, testosterone was positively associated with acute socio-emotional stress (*b* = 37.34, *p*_*FDR*_ = 0.0456), which, however, was not the case for the other two groups (IUD: *b* = −28.07, *p* = 0.202; OC = −20.79, *p* = 0.37; Fig. 4(b)). In IUD users, oestradiol negatively predicted negative affect (*b* = −3.38, *p*_*fdr*_ = 0.01). In OC users, this association was also negative but not significant (*b* = −0.69, *p* = 0.839), while in NC women, it was slightly positive (*b* = 0.79, *p* = 0.491), although not significant (Fig. 4(c)). No other association between the endogenous and exogenous (LNG, EE) sex hormones and the stress correlates or 7-day diary variables emerged (all *p*s > 0.05).

## Discussion

The present study investigated multidimensional stress responses in women using hormonal IUDs and OCs and NC women in the mid-luteal phase at two timepoints. To the best of our knowledge, it is the first study to investigate the association between IUD use and stress responses in a repeated manner, while also assessing exogenous hormone levels. Stress was successfully induced in all groups of women, as indicated by subjective ratings, cortisol and cortisone responses, heart rate and skin conductance. Women using hormonal contraceptives differed on distinct dimensions in comparison to NC women: IUD users exhibited elevated subjective stress, negative affect and anxiety and lower positive affect compared with NC women. OC users showed a blunted salivary cortisol and cortisone response to stress compared to both other groups and a stress-independent heightened heart rate. Using the 7-day diary, OC users reported less acute stress and fewer negative emotions than either IUD users or NC women.

### Increased emotional but not physiological stress response in IUD users

Women using IUDs reported higher negative affect after stress induction than the other two groups, and more subjective stress and anxiety and lower positive affect after stress than NC women, indicating an increased negative response to stress. This trend continued in the 7-day diary, in which IUD users reported more negative affect and emotions, acute work stress, anger and sadness than OC users. Women using IUDs also reported the highest current affect of life stressors, although they did not experience more life stressors than the other groups. Hence, our data suggest that IUD users have a higher emotional reactivity following stress induction both in the laboratory and in their daily lives over the following days. IUD users seem to have a higher subjective susceptibility to stressors and report a more negative stress experience compared with NC women and OC users. Zelionkaitė et al^8^ found that IUD users, compared with OC users and NC women, showed a higher N2 amplitude in event-related potentials (ERPs) when instructed to upregulate negative emotions, suggesting greater recruitment of attention and cognitive control. This increased engagement during emotion regulation may also contribute to increased subjective stress in IUD users in our study. Notably, this heightened emotional response is not reflected in any of the physiological parameters, including the cortisol and cortisone responses. This contrasts with the only other study that investigated stress responses in women using IUDs: Aleknaviciute et al^7^ reported a potentiated cortisol response and increased heart rate in IUD users compared to NC women and copper IUD users. These discrepancies might be explained by a dose–response relationship, as the previous study only included women using the high-dose IUD. Our sample consisted mainly of women using lower-dose preparations, with only one participant using the high-dose IUD (Supplement Table S1). Presumably, the higher dosage of LNG could lead to a higher cortisol response. In line with this, results from a recent Danish registry study showed a higher risk of antidepressant prescription with the high-dose compared to the low-dose LNG-IUD.^12^ In addition, women who use progestin-only OCs (which contain higher concentrations of progestins than IUDs) seem at higher risk of mental health symptoms than women who do not use them,^27^ suggesting that progestin-only hormonal contraceptives need to be investigated, taking into account their dosage.

### Blunted cortisol, lower 7-day diary stress but increased heart rate in women using OCs

Women using OCs in our sample exhibited a blunted cortisol and cortisone stress response, as previously indicated.^5,7^ Interestingly, OC users also had the highest serum cortisol levels. This paradox can be explained by the role of cortisol binding globulin (CBG), which is increased by EE and binds free cortisol.^28^ Consequently, the blunted cortisol response in OC users may result from the binding of cortisol by CBG, rendering it inactive and undetectable in saliva, while serum measures reflect both bound and unbound cortisol. When controlling for CBG levels, total serum cortisol did not significantly differ among women using IUDs, OCs and NC women after adrenocorticotropic hormone (ACTH) administration.^7^ Thus, assessing CBG levels in future hormonal contraception studies is essential to better understand how hormonal contraceptives affect stress responses.

The stress-independent heart rate increase among OC users aligns with data indicating a slightly higher heart rate with OC use in 24-h measurements.^29,30^ EE is known to increase angiotensin II, leading to increased blood pressure^31^ and possibly affecting heart rate.^32^ Contrary to the increased physiological responses, women using OCs reported the lowest negative emotions, acute work and socio-emotional stress in the 7-day diary compared with the two other groups. The assessment of NC women in their premenstrual phase and differing hormonal environments may explain these differences, with OC use presumably stabilising mood in daily life.^33^

### Endogenous and exogenous sex hormones influence stress responses

This study is the first to report on both exogenous and endogenous hormones in relation to the stress response. Few studies have examined exogenous hormones and affect processing,^25,34–36^ although the hormonal contraception literature suggests interactions of exogenous and endogenous sex hormones with stress.^5^ Our exploratory analyses for exogenous and endogenous hormones revealed no direct association of the exogenous (EE, LNG) hormones with stress correlates or 7-day diary variables. Progesterone negatively predicted skin conductance during acute stress, consistent with its role in reducing postmenopausal symptoms such as night sweats.^37^ Nevertheless, a positive correlation with skin conductance during exposure to negative images found in another study^38^ suggests a complex relationship potentially influenced by cyclical variations in bound progesterone.^39^ Testosterone was positively predictive for acute socio-emotional stress in the 7-day diary among NC women, suggesting a link with social competition,^40^ whereas oestradiol negatively predicted negative affect in the 7-day diary among IUD users, potentially indicating a protective effect of oestradiol on mood through modulation of and interaction with various neurotransmitter pathways.^41,42^

In our sample, exogenous hormones were EE for OC users and the progestin LNG for IUD users and more than half of OC users. As our data show, the use of exogenous hormones affects endogenous hormone levels (Fig. 2). Oestradiol and progesterone were differently suppressed in OC and IUD users when compared with NC women. EE can upregulate sex hormone binding globulin (SHBG) (binding oestrogens and androgens^43^) and CBG (binding cortisol and progesterone^44^) as well as progesterone receptors, providing more progesterone receptors for circulating progesterone to act upon.^45^ Progestins can bind to progesterone receptors and other steroid receptors and competitively inhibit or activate them to different extents.^46^ Importantly, serum endogenous hormone levels reflect both bound (to SHBG/CBG) and unbound quantities^43^ and exogenous hormones can be bound with varying affinities, highlighting the need to measure SHBG and CBG levels in future stress research. These explanations illustrate how hormonal contraception effects on stress result from a complex interaction of both endogenous and exogenous sex hormones and their bioavailability.

Although direct associations of stress correlates with exogenous hormones were not found in our sample, the inclusion of both endogenous and exogenous hormones may elucidate the interaction of sex hormones with the HPA axis and stress processing brain areas with high steroid receptor densities^47^ that influence stress response. The differential effects of combined exogenous hormone administration on endogenous hormone levels and stress in our data suggest systemic and mechanistic influences that warrant further investigation. In addition, individual physiological and subjective sensitivities to sex hormone variations may explain the observed differences in stress responses and should be considered in future research.

### Methodological considerations

As strengths, the current study assessed multiple stress correlates to disentangle the impact of hormonal contraception on cortisol and cortisone and physiological and subjective stress responses, as well as their association with exogenous and endogenous sex hormones. The longitudinal study design (two repeated measurements) confirmed the robustness of stress responses, specifically cortisol and subjective stress. Physiological measures of heart rate and skin conductance are more susceptible to habituation, which has also been shown previously for heart rate,^48^ and should be considered in future research. Levels of testosterone, progesterone and LNG were higher at the second timepoint, which is to be expected, as sex hormones have high inter-individual variability^49^ and anticipation of a challenging situation may lead to higher testosterone levels^50^ at the second measurement.

Future research should consider analyses based on the classification of progestins, taking into account their (anti-)estrogenic and androgenic properties.^46,51^ The sample size of our study limited such analyses but assessing the steroid receptor affinity of different progestins may clarify the effects of hormonal contraception on stress responses. Because of irregular bleeding, determination of the cycle phase in IUD users was not feasible and potentially introduced additional variability into the data, as most women using IUDs still ovulate.^52^ In addition, LNG-IUDs are often chosen by women with gynaecological and/or mood-related problems (e.g. endometriosis, menorrhagia, adverse effects of oral contraceptive use^9^). Although we excluded women with known mental health or gynaecological disorders, subclinical or undiagnosed symptoms could confound our results. Therefore, future research should not only assess the reason for use and cycle phase in IUD users but also extend this research to clinical populations.^53^

Our sample consisted of heterosexual, cisgender women (students from Germany). To generalise the results to a broader, more diverse population, future studies should include a more diverse sample.

### Implications

In this study, stress responses were repeatedly assessed in women using hormonal IUDs and OCs and NC women. The results indicate that IUDs and OCs affect stress responses differently, providing initial insights into the mechanisms of exogenous, locally administered, sex hormones. Women using IUDs were more susceptible to subjective stress without alterations in cortisol response, whereas women using OCs showed a blunted salivary cortisol stress response but stress-independent higher levels of serum cortisol and heart rate. The increased susceptibility to stress and negative mood in IUD users warrants further investigation, especially in light of recent reports of associations between IUD use and depression.^11,12^ Future research should explore the vulnerabilities related to natural hormonal fluctuations and the oral and intrauterine administration of exogenous hormones. This research could clarify the higher prevalence rates of stress-related disorders such as depression and anxiety in women using hormonal contraceptives.

## Supporting information

Bürger et al. supplementary material Bürger et al. supplementary material

## Data Availability

The data, analytic code and research material that support the findings of this study are available from the corresponding author, B.D., upon reasonable request.

## Supplementary material

The supplementary material for this article can be found at https://doi.org/10.1192/bjp.2025.7

## References

[1] Heck AL , Handa RJ. Sex differences in the hypothalamic–pituitary–adrenal axis’ response to stress: an important role for gonadal hormones. Neuropsychopharmacology 2019; 44: 45. 30111811 10.1038/s41386-018-0167-9 PMC6235871

[2] Smeets T , Cornelisse S , Quaedflieg CWEM , Meyer T , Jelicic M , Merckelbach H. Introducing the Maastricht Acute Stress Test (MAST): a quick and non-invasive approach to elicit robust autonomic and glucocorticoid stress responses. Psychoneuroendocrinology 2012; 37: 1998–2008. 22608857 10.1016/j.psyneuen.2012.04.012

[3] Spencer RL , Deak T. A users guide to HPA axis research. Physiol Behav 2017; 178: 43–65. 27871862 10.1016/j.physbeh.2016.11.014 PMC5451309

[4] Kirschbaum C , Kudielka BM , Gaab J , Schommer NC , Hellhammer DH. Impact of gender, menstrual cycle phase, and oral contraceptives on the activity of the hypothalamus-pituitary-adrenal axis. Psychosomat Med 1999; 61: 154–62. 10.1097/00006842-199903000-00006 10204967

[5] Gervasio J , Zheng S , Skrotzki C , Pachete A. The effect of oral contraceptive use on cortisol reactivity to the Trier Social Stress Test: a meta-analysis. Psychoneuroendocrinology 2022; 136: 105626. 34922094 10.1016/j.psyneuen.2021.105626

[6] United Nations. Contraceptive Use by Method 2019: Data Booklet. UN, 2019 (10.18356/1bd58a10-en).

[7] Aleknaviciute J , Tulen JHM , De Rijke YB , Bouwkamp CG , van der Kroeg M , Timmermans M , et al. The levonorgestrel-releasing intrauterine device potentiates stress reactivity. Psychoneuroendocrinology 2017; 80: 39–45. 28315609 10.1016/j.psyneuen.2017.02.025

[8] Zelionkaitė I , Gaižauskaitė R , Uusberg H , Uusberg A , Ambrasė A , Derntl B , et al. The levonorgestrel-releasing intrauterine device is related to early emotional reactivity: an ERP study. Psychoneuroendocrinology 2024; 162: 106954. 38241970 10.1016/j.psyneuen.2023.106954

[9] Bürger Z , Magdalena Bucher A , Comasco E , Henes M , Hübner S , Kogler L , et al. Association of levonorgestrel intrauterine devices with stress reactivity, mental health, quality of life and sexual functioning: a systematic review. Front Neuroendocrinol 2021; 63: 100943. 34425187 10.1016/j.yfrne.2021.100943

[10] Elsayed M , Dardeer KT , Khehra N , Padda I , Graf H , Soliman A , et al. The potential association between psychiatric symptoms and the use of levonorgestrel intrauterine devices (LNG–IUDs): a systematic review. World J Biol Psychiatry 2023; 6: 457–75. 10.1080/15622975.2022.2145354 36426589

[11] Stenhammar E , Wikman P , Gemzell Danielsson K , Kopp-Kallner H , Sundström Poromaa I. Levonorgestrel intrauterine device and depression: a Swedish register-based cohort study. Int J Psychophysiol 2023; 193: 112230. 37611669 10.1016/j.ijpsycho.2023.08.003

[12] Larsen SV , Mikkelsen AP , Ozenne B , Munk-Olsen T , Lidegaard Ø , Frokjaer VG. Association between intrauterine system hormone dosage and depression risk. Am J Psychiatry 2024; 181: 834–41. 38982827 10.1176/appi.ajp.20230909

[13] Faul F , Erdfelder E , Lang A-G , Buchner A. G*Power 3: a flexible statistical power analysis program for the social, behavioral, and biomedical sciences. Behav Res Methods 2007; 39: 175–91. 17695343 10.3758/bf03193146

[14] Heidari S , Babor TF , De Castro P , Tort S , Curno M. Sex and gender equity in research: rationale for the SAGER guidelines and recommended use. Res Integ Peer Rev 2016; 1: 2. 10.1186/s41073-016-0007-6 PMC5793986 29451543

[15] *Nature Medicine*. Editorial: a life-course approach to women’s health. Nat Med 2024; 30: 1–1. Available from: 10.1038/s41591-023-02777-8. 38242978

[16] Kühner C , Bürger C , Keller F , Hautzinger M. Reliabilität und Validität des revidierten Beck-Depressionsinventars (BDI-II). [Reliability and validity of the revised Beck Depression Inventory (BDI-II).] Nervenarzt 2007; 78: 651–6. 16832698 10.1007/s00115-006-2098-7

[17] Laux L. Das State-Trait-Angstinventar (STAI): theoretische Grundlagen und Handanweisung. [*The State-Trait Anxiety Inventory (STAI): Theoretical Principles and Manual*.] Beltz, 1981 (https://fis.uni-bamberg.de/handle/uniba/26756).

[18] Wingenfeld K , Spitzer C , Mensebach C , Grabe HJ , Hill A , Gast U , et al. The German Version of the Childhood Trauma Questionnaire (CTQ): preliminary psychometric properties. Psychother Psychosom Med Psychol 2010; 60: e13. 20361390 10.1055/s-0030-1253494

[19] Ungerer O , Deter H-C , Fikentscher E , Konzag TA. Improved diagnostics of trauma-related disease through the application of the Life-Stressor Checklist. Psychother Psychosom Med Psychol 2010; 60: 434–41. 20200805 10.1055/s-0030-1247497

[20] Angermeyer MC , Kilian R , Matschinger H . WHOQOL – 100 und WHOQOL – BREF Handbuch für die deutschsprachige Version der WHO-Instrumente zur Erfassung von Lebensqualität. [*WHOQOL – 100 and WHOQOL – BREF Manual for the German-Language Version of the WHO Quality of Life Instruments*.] Hogrefe, 2000 (https://d-nb.info/959636390/04).

[21] Krohne HW , Egloff B , Kohlmann C-W , Tausch A. Positive and Negative Affect Schedule – German Version (PANAS) [Database Record]. APA PsycTests, 1996 (10.1037/t49650-000).

[22] Schneider F , Gur RC , Gur RE , Muenz LR. Standardized mood induction with happy and sad facial expressions. Psychiatry Res 1994; 51: 19–31. 8197269 10.1016/0165-1781(94)90044-2

[23] Aydin E , Drotleff B , Noack H , Derntl B , Lämmerhofer M. Fast accurate quantification of salivary cortisol and cortisone in a large-scale clinical stress study by micro-UHPLC-ESI-MS/MS using a surrogate calibrant approach. J Chromatogr B 2021; 1182: 122939. 10.1016/j.jchromb.2021.122939 34547590

[24] Pruessner JC , Kirschbaum C , Meinlschmid G , Hellhammer DH. Two formulas for computation of the area under the curve represent measures of total hormone concentration versus time-dependent change. Psychoneuroendocrinology 2003; 28: 916–31. 12892658 10.1016/s0306-4530(02)00108-7

[25] Kimmig A-CS , Wildgruber D , Gärtner A , Drotleff B , Krylova M , Lämmerhofer M , et al. Lower affective empathy in oral contraceptive users: a cross-sectional fMRI study. Cereb Cortex 2023; 33: 4319–33. 36137568 10.1093/cercor/bhac345

[26] Rodriguez LA , Casey E , Crossley E , Williams N , Dhaher YY. The hormonal profile in women using combined monophasic oral contraceptive pills varies across the pill cycle: a temporal analysis of serum endogenous and exogenous hormones using liquid chromatography with tandem mass spectroscopy. Am J Physiol Endocrinol Metabol 2024; 327: E121–33. 10.1152/ajpendo.00418.2023 PMC11390121 38775726

[27] Kraft MZ , Rojczyk P , Weiss T , Derntl B , Kikinis Z , Croy I , et al. Symptoms of mental disorders and oral contraception use: a systematic review and meta-analysis. Front Neuroendocrinol 2024; 72: 101111. 37967755 10.1016/j.yfrne.2023.101111

[28] Kumsta R , Entringer S , Hellhammer DH , Wüst S. Cortisol and ACTH responses to psychosocial stress are modulated by corticosteroid binding globulin levels. Psychoneuroendocrinology 2007; 32: 1153–7. 17904296 10.1016/j.psyneuen.2007.08.007

[29] Reinberg AE , Touitou Y , Soudant E , Bernard D , Bazin R , Mechkouri M. Oral contraceptives alter circadian rhythm parameters of cortisol, melatonin, blood pressure, heart rate, skin blood flow, transepidermal water loss, and skin amino acids of healthy young women. Chronobiol Int 1996; 13: 199–211. 8874983 10.3109/07420529609012653

[30] Cagnacci A , Ferrari S , Napolitano A , Piacenti I , Arangino S , Volpe A. Combined oral contraceptive containing drospirenone does not modify 24-h ambulatory blood pressure but increases heart rate in healthy young women: prospective study. Contraception 2013; 88: 413–7. 23312932 10.1016/j.contraception.2012.12.002

[31] Oelkers WKH. Effects of estrogens and progestogens on the renin-aldosterone system and blood pressure. Steroids 1996; 61: 166–71. 8732994 10.1016/0039-128x(96)00007-4

[32] Cagnacci A , Zanin R , Napolitano A , Arangino S , Volpe A. Modification of 24-h ambulatory blood pressure and heart rate during contraception with the vaginal ring: a prospective study. Contraception 2013; 88: 539–43. 23683580 10.1016/j.contraception.2013.04.003

[33] Oinonen KA , Mazmanian D. To what extent do oral contraceptives influence mood and affect? J Affect Disord 2002; 70: 229–40. 12128235 10.1016/s0165-0327(01)00356-1

[34] Kimmig A-CS , Friedrich P , Drotleff B , Lämmerhofer M , Sundström-Poromaa I , Weis S , et al. To start or to discontinue the pill – changes in progestogens reflected by resting-state connectivity and positive mood. *BioRxiv* [Preprint] 2022. Available from: 10.1101/2022.09.21.508780.

[35] Kimmig A-CS , Bischofberger JA , Birrenbach AD , Drotleff B , Lämmerhofer M , Sundström-Poromaa I , et al. No evidence for a role of oral contraceptive-use in emotion recognition but higher negativity bias in early follicular women. Front Behav Neurosci 2022; 15: 773961. 35126066 10.3389/fnbeh.2021.773961 PMC8814336

[36] Brouillard A , Davignon L-M , Turcotte A-M , Marin M-F. Morphologic alterations of the fear circuitry: the role of sex hormones and oral contraceptives. Front Endocrinol 2023; 14: 1228504. 10.3389/fendo.2023.1228504 PMC10661904 38027091

[37] Prior JC. Progesterone for treatment of symptomatic menopausal women. Climacteric 2018; 21: 358–65. 29962247 10.1080/13697137.2018.1472567

[38] Gamsakhurdashvili D , Antov MI , Stockhorst U. Sex-hormone status and emotional processing in healthy women. Psychoneuroendocrinology 2021; 130: 105258. 34058558 10.1016/j.psyneuen.2021.105258

[39] Evans JJ. Progesterone in saliva does not parallel unbound progesterone in plasma. Clin Chem 1986; 32: 542–4. 3948403

[40] Casto KV , Edwards DA . Testosterone, cortisol, and human competition. Hormones Behav 2016; 82: 21–37. 10.1016/j.yhbeh.2016.04.004 27103058

[41] Lokuge S , Frey BN , Foster JA , Soares CN , Steiner M. Depression in women: windows of vulnerability and new insights into the link between estrogen and serotonin. J Clin Psychiatry 2011; 72: 3297. 10.4088/JCP.11com07089 22127200

[42] Del Río JP , Alliende MI , Molina N , Serrano FG , Molina S , Vigil P. Steroid hormones and their action in women’s brains: the importance of hormonal balance. Front Public Health 2018; 6: 141. 29876339 10.3389/fpubh.2018.00141 PMC5974145

[43] Iqbal MJ , Dalton M , Sawers RS. Binding of testosterone and oestradiol to sex hormone binding globulin, human serum albumin and other plasma proteins: evidence for non-specific binding of oestradiol to sex hormone binding globulin. Clin Sci 1983; 64: 307–14. 10.1042/cs0640307 6681600

[44] Dunn JF , Nisula BC , Rodbard D. Transport of steroid hormones: binding of 21 endogenous steroids to both testosterone-binding globulin and corticosteroid-binding globulin in human plasma. J Clin Endocrinol Metab 1981; 53: 58–68. 7195404 10.1210/jcem-53-1-58

[45] Sundström-Poromaa I , Comasco E , Sumner R , Luders E. Progesterone – friend or foe? Front Neuroendocrinol 2020; 59: 100856. 32730861 10.1016/j.yfrne.2020.100856

[46] Birkhäuser M. Klinische Bedeutung von gestagenen Partialwirkungen. [Clinical significance of progestogen partial effects.] Gynäkol Endokrinol 2006; 4: 52–64.

[47] McEwen BS , Milner TA. Understanding the broad influence of sex hormones and sex differences in the brain. J Neurosci Res 2017; 95: 24–39. 27870427 10.1002/jnr.23809 PMC5120618

[48] Bullock T , MacLean MH , Santander T , Boone AP , Babenko V , Dundon NM , et al. Habituation of the stress response multiplex to repeated cold pressor exposure. Front Physiol 2023; 13: 752900. 36703933 10.3389/fphys.2022.752900 PMC9871365

[49] Schmalenberger KM , Tauseef HA , Barone JC , Owens SA , Lieberman L , Jarczok MN , et al. How to study the menstrual cycle: Practical tools and recommendations. Psychoneuroendocrinology 2021; 123: 104895. 33113391 10.1016/j.psyneuen.2020.104895 PMC8363181

[50] Khosravi S , Kogler L , Khosrowabadi R , Hashemi T , Derntl B , Heysieattalab S. The influence of endogenous and exogenous sex steroid hormones and social hierarchy on decision-making: a systematic review. [Preprint] 2023. Available from: 10.21203/rs.3.rs-3589121/v1. 42025391

[51] Dickey RP. Managing Contraceptive Pill Patients. EMIS, Medical Publishers, 2014 (http://archive.org/details/managingcontrace0000dick).

[52] Xiao B , Zeng T , Wu S , Sun H , Xiao N. Effect of levonorgestrel-releasing intrauterine device on hormonal profile and menstrual pattern after long-term use. Contraception 1995; 51: 359–65. 7554977 10.1016/0010-7824(95)00102-g

[53] Bale TL , Epperson CN. Sex differences and stress across the lifespan. Nature Neurosci 2015; 18: 1413. 26404716 10.1038/nn.4112 PMC4620712

