# Supplementary material for: Subjective, behavioural and physiological correlates of stress in women using hormonal contraceptives
Source: Br J Psychiatry. 2025 Jun 13;226(6):392–400. doi: 10.1192/bjp.2025.7 (PMC12257283; doi:10.1192/bjp.2025.7)
Supplement: Bürger et al. supplementary material [file S0007125025000078sup001.pdf]

Supplement for the manuscript “Subjective, behavioural and physiological correlates of stress in women using hormonal contraceptives” by Bürger et al.

Table of content

Supplementary Methods 3
- In- and Exclusion Criteria 3
- Supplement Table S1. Oral contraceptive and IUD composition 4
- Maastricht Acute Stress Task 5
- General self-report questionnaires 5
- Task-related subjective stress and affect 5
- Steroid hormones in plasma & Salivary Cortisol/Cortisone 6
- Physiological stress 7
- Statistical Analysis 7

Supplementary Results 8
- Study Population 8
- Supplement Table S2. Sample description 8
- Steroid hormones in plasma 9
- Supplement Table S3. Steroid hormones 11
- Supplement Table S4. Steroid hormone references values 12
- Repeated measures matter for ANS but not cortisol or subjective stress 13
- Successful Stress Induction 13
- Subjective stress altered in IUDs, cortisol in OCs 13
- Daily Diary shows lower negative affect and stress in OCs 14

Supplement Table S5. 7-day-diary variables and statistics. 14

Sex hormones affect stress response 15

Supplementary Tables (referenced in manuscript) 16

Supplement Table S6. Number of women for each task presentation order 16

Supplement Table S7. 7-day-diary variables....16

Supplementary Figures (referenced in manuscript) 18

Supplement Figure S1. Cortisol AUCi....18

Supplement Figure S2. Cortisone 18

Supplement Figure S3. Cortisol-to-cortisone ratio....19

References....20

Supplementary Methods

In- and Exclusion Criteria

NC women had a regular menstrual cycle and were measured during the mid-luteal phase (day +6 to +10 after positive LH-test[1]). Cycle phase was determined using ovulation tests (self diagnostics LH tests, nal von minden GmbH, Germany) and confirmed by backwards counting and hormonal levels. OC-users took monophasic combined OCs containing EE with any kind of progestin, apart from one participant with a multiphasic OC with oestradiol valerate and a progestin (measured during highest progestin dosage). IUD-users used one of the three LNG-IUDs available in Germany. For details on OC and IUD type, see Supplement Table S1 below.

We accounted for sex hormone changes by following guidelines for menstrual cycle assessment[1–3], by measuring OC-users during their active pill intake, and IUD-users outside their menstruation. All women were healthy, with a BMI between 18-25 kg/m2, non-smokers, white, fluent in German and had at least an advanced technical college entrance qualification. Considering the impact of sexual orientation on cortisol response[4], only heterosexual women were included. We screened for neurological and mental disorders based on standardised diagnoses via a structured clinical interview (SCID[5]). Women with mental, chronic, hormonal, or metabolic disorders were excluded. Women having been pregnant or breastfeeding in the last year were excluded, as well as women using other hormonal treatment besides OC or IUD. Premenstrual mood changes were documented in all women at each measurement day using the Premenstrual Symptoms Screening Tool (PSST[6]) and women with premenstrual dysphoric disorder were excluded. Shift workers[7] and competitive athletes[8] were excluded due to the known impact on cortisol response.

Supplement Table S1. Oral contraceptive and IUD composition

**Oral contraceptive composition**					
**Progestin**	**Quantity (µg)**	**Exogenous oestradiol**	**Quantity (µg)**	**Number of participants**	**Brand name**
Chlormadinone acetate[1,3,4]	2000	Ethinyl oestradiol	30 µg	4	Solera®, Belara®, Belissima®, Lilia®
Cyproterone acetate[1,3,4]	2000	Ethinyl oestradiol	35	1	Juliette®
Dienogest[1,3]	2000	Ethinyl oestradiol	30	7	Maxim®, Sibilla®, Dienovel®
Dienogest[1,3]	3000	Oestradiol valerate	2000	1	Qlaira® (multiphasic, measured on these quantities)
Levonorgestrel[1,3]	100	Ethinyl oestradiol	20	11	Leona® Hexal®, Evaluna 20®, Swingo 20®, Asumate 20®
Levonorgestrel[1,3]	125	Ethinyl oestradiol	30	1	Maexeni 30 mite®
Levonorgestrel[1,3]	150	Ethinyl oestradiol	30	4	Levomin 30®, Swingo 30®, Asumate 30®
Norgestimate[1,4(?),5(?)]	250	Ethinyl oestradiol	35	1	Amicette®
**IUD composition**					
**Progestin**	**Quantity (µg/24h) ***			**Number of participants**	**Brand Name**
Levonorgestrel[1]	6		0	8	Jaydess®
Levonorgestrel[1]	9		0	18	Kyleena®
Levonorgestrel[1]	14		0	1	Mirena®

Note.[1]=anti-estrogenic; [2]=androgenic; [3]=anti-androgenic; [4]=glucocorticoid; [5]=anti-mineralocorticoid, [?]=unknown (from Gobrecht-Keller[9]); *mean release rate over 3 (Jaydess®) or 5 (Kyleena® & Mirena®) years

Maastricht Acute Stress Task

In this ten-minute task[10], preceded by a five-minute preparation, participants alternate putting their hand in ice water (approx. 7°C) and doing mental arithmetic calculations (i.e. calculating from 1958 in steps of 17). In total, five trials of cold water (max. 90 s) and four trials of mental arithmetic calculations (min. 45 s) are performed, combining physiological stress with achievement stress. During the task, an experimenter in a lab coat gives neutral to negative feedback and participants are videotaped to induce additional social stress. In the placebo condition, trials with lukewarm water (approx. 30°C) are alternated with counting (from 1 to 25). No videotaping or feedback from an experimenter is given. The amount and duration of trials in the placebo condition are the same as in the stress condition.

General self-report questionnaires

We assessed depressive symptoms (Beck's Depression Inventory, BDI-II[11]), trait anxiety (State and Trait Anxiety Inventory, STAI-T, [12]), childhood trauma and life stressors (Childhood Trauma Questionnaire, CTQ[13]; revised Life Stressor Checklist, LSC-R[14]), overall quality of life (WHO Quality of Life questionnaire, WHOQOL[15]), self-esteem (Rosenberg Self-Esteem Scale, RSES[16]), chronic and ongoing stress (Trier Inventory for Chronic Stress, TICS[17]; Perceived Stress Scale, PSS-10[18]), stress coping mechanisms (Coping Inventory for Stressful Situations, CISS[19]), emotional regulation mechanisms (Emotion Regulation Questionnaire, ERQ[20]), sexual function (Female Sexual Function Index, FSFI[21]), premenstrual syndrome (PMS) and premenstrual dysphoric disorder (PMDD) (Premenstrual Symptoms Screening Tool, PSST[6]).

Task-related subjective stress and affect

The questionnaire used were the Positive and Negative Affect Scale (PANAS[22]), State and Trait Anxiety Inventory (state only, STAI-S[12]) and emotional scale rating (ESR[23]).

Steroid hormones in plasma & Salivary Cortisol/Cortisone

Hormone levels of testosterone, progesterone, oestradiol, cortisol, EE and LNG were assessed in blood. Additionally, dihydrotestosterone, pregnenolone, allopregnanolone, hydroxyprogesterone, oestrone, oestriol, corticosterone, cortisone and 4 additional progestins (dienogest, norgestimate, cyproterone acetate, chlormadinone acetate) were measured. Blood drawn was centrifuged for 15 min at 4400 rpm. Surface plasma was aliquoted into 1.5 mL tubes and stored at –80°C until analysis. The analytical system consisted of a 1290 Infinity II UHPLC (Agilent Technologies, Germany) coupled to a QTRAP 4500 mass spectrometer (Sciex, United States). The hormones were quantified via a surrogate calibrant approach (Li and Cohen, 2003; Drotleff et al., 2018) and the method was validated according to FDA guidelines. The dynamic range of oestrogens, progesterone, testosterone, EE and the various progestins ranged from 3.54–5685.65, 1.0–61450.13, 1.97–9847.31, 2.0–3200, and 10–20000 pg/mL, respectively. To evaluate the performance of the method and document the validity of the analytical measurement methods, quality control samples (QCs) were analysed on three consecutive days. Interday precision (i.e., repeatability between different days) and accuracy (as % recovery of QCs’ nominal concentration) were 0.06–9.55% and 85.29–109.31% (oestrogens), 3.63–8.93 and 88.12–111.56% (progesterones), 3.50–7.88% and 89.24–111.63% (testosterone), 0.06–3.57% and 85.29–109.31% (EE), as well as 0.26-8.88% and 85.53–109.04% (progestins), indicating excellent method performance within the acceptance criteria of the FDA bioanalytical method validation guideline. Interday precision measures the repeatability of the concentrations of the quality control samples between different days and interday accuracy the percent recovery (% found/nominal concentration) in the quality control samples on the different days.

Saliva samples were frozen immediately after collection and stored at −20°C until analysis. Samples were prepared for analysis in several steps, including slow thawing at 4°C, protein precipitation, solid-phase extraction on a 96-well plate Oasis PRIME hydrophilic–lipophilic balance material, drying under nitrogen, and reconstitution by 50 µL of MeOH-H2O (30:70; v/v) in

a 96-well sealed collection plate. Chromatographic separation was performed on a micro-LC instrument (MicroLC 200 Plus, Sciex, Framing-ham, MA, USA), and analyte detection was carried out on a hyphenated triple-quadrupole mass-spectrometer (QTRAP 4500, Sciex) in negative ionization mode. For absolute quantification, a surrogate calibrant method using Cortisol-d4 and Cortisone-13C3 in true saliva matrix was established. Internal standardization was obtained by spiking of cortisone-d8. Linear ranges for cortisol and cortisone were in the range from 0.072 to 44 ng/mL for Cortisone-13C3 and 0.062 to 75.5 ng/mL Cortisol-d4, respectively. The method was validated according to FDA guidelines.

Physiological stress

Recording of heart-rate (HR) and skin-conductance (SC) was done using a BIOPAC MP160 with a BioNomadix Wireless Transmitter and the AcqKnowledge5 software (all Biopac Systems, Inc. Goleta, CA, USA). HR was measured using a photoplethysmogram (PPG) sensor on the index finger, SC was measured using SC electrodes (BIOPAC EL507) on the middle phalanges of the dominant hand. Measurement was continuous from the beginning of the first relaxation to the end of the third relaxation period. HR was further processed and analysed using Kubios HRV Scientific 4.0.2 (Kubios Oy, Kuopio, Finland). SC was processed and analysed with Ledalab (V.3.4.9, ledalab.de[24]) implemented in Matlab R2023a (The Mathworks Inc., Natick, MA, USA).

Statistical Analysis

The following R packages were used for analysis and data visualisation: *haven, readxl, tidyverse, rstatix, multcomp, lmerTest, psych, effectsize, texreg, easystats and ggpubr*. The *performance* package was used to verify assumptions of statistical models.

Supplementary Results

Study Population

A total of 88 participants were recruited. After exclusion (high BMI, abortion for pain reasons (2 OCs)), 86 participants (29 NC; 30 OC; 27 IUD) were included for the first measurement, and 75 (25 NC; 25 OC; 25 IUD) for the second measurement four months later (121±37.4 days). NC-women had an average cycle duration of 29.7±5.39 days during both measurement periods and were assessed on average 6.96 days before menstruation in the mid-luteal phase. OC-users were measured during active pill intake (days 2-21; multiphasic OC-user: day 9-24, highest dose of progestin). Seventeen OC-users used an androgenic and thirteen an anti-androgenic OC. In the IUD-group, eight women used the 13.5mg-LNG-IUD, eighteen the 19.5mg-LNG-IUD and one the 52mg-LNG-IUD (detailed HC composition in supplement Table S1 above). Full sample description in supplement Table S2 below.

Supplement Table S2. Sample description

Values indicate means and SD in parentheses.

	IUD (n=27)	OC (n=30)	NC (n=29)	Total (n=86)	Statistic	p-value	Eta squared
**Age (years)**	24.15 (3.27)	22.67 (2.52)	24.07 (2.25)	23.60 (2.75)	F(2, 83) = 2.79	0.067	0.06
**Depression score (BDI-II[11])**	3.37 (4.26)	3.00 (3.75)	2.31 (3.23)	2.88 (3.74)	F(2, 83) = 0.58	0.563	0.01
**Trait anxiety (STAI-T[12])**	33.96 (6.35)	33.07 (4.32)	34.31 (8.76)	33.77 (6.66)	F(2, 83) = 0.27	0.764	0.006
**Childhood trauma (CTQ[13])**	34.30 (4.26)	34.67 (4.76)	34.48 (5.53)	34.49 (4.83)	F(2, 83) = 0.04	0.960	0.001
**Emotion regulation via reappraisal (ERQ[20])**	3.76 (0.76)	3.56 (0.61)	3.56 (0.58)	3.62 (0.65)	F(2, 83) = 0.88	0.419	0.02

**Emotion regulation via suppression (ERQ[20])**	2.51 (0.61)	2.35 (0.73)	2.40 (0.74)	2.42 (0.69)	F(2, 83) = 0.39	0.681	0.009
**Self-esteem (RSES[16])**	40.89 (4.56)	40.23 (4.34)	40.38 (4.17)	40.49 (4.31)	F(2, 83) = 0.17	0.840	0.004
**Overall quality of life (WHOQOL[15])**	17.33 (2.77)	17.53 (2.15)	17.66 (2.62)	17.51 (2.49)	F(2, 83) = 0.12	0.891	0.003
**Life stressors sum (LSC-R[14])**	2.48 (1.76)	2.10 (1.40)	1.97 (1.35)	2.17 (1.50)	F(2, 83) = 0.88	0.420	0.02
**Life stressors weighted for current impact (LSC-R[14])**	6.30 (5.86)	4.57 (3.61)	3.38 (2.69)	4.71 (4.33)	F(2, 83) = 3.38	***0.039****	0.08
**Chronic stress (TICS[17])**	25.30 (6.93)	23.60 (6.69)	24.38 (7.45)	24.40 (6.98)	F(2, 83) = 0.41	0.662	0.010
**Perceived stress (PSS-10[18])**	24.81 (5.55)	23.83 (5.05)	24.55 (6.17)	24.38 (5.55)	F(2, 83) = 0.24	0.789	0.006
**PMS (n)**	4	4	3	11	$\chi^2(2)$ = 0.26	0.877	-
**Relationship status (single/relationship/married)**	9/16/2	1/29/0	14/13/2	24/58/4	$\chi^2(4)$ = 19.51	**<.001***	-

Notes. PMS: premenstrual syndrome. * indicates significant difference at p=.05.

Steroid hormones in plasma

Statistically significant outcome values for steroid hormones

- Testosterone
  - T1 vs. T2: b = 0.40, 95% CI [0.22, 0.57], t(146) = 4.46, p < .001
- Dihydrotestosterone
  - NC vs. IUD: b = -0.56, 95% CI [-0.93, -0.19], t(148) = -3.02, p = 0.003
  - NC vs OC: b = -0.41, 95% CI [-0.77, -0.06], t(148) = -2.30, p = 0.023
- Progesterone
  - NC vs. IUD: b = -2.22, 95% CI [-3.00, -1.44], t(153) = -5.61, p < .001
  - NC vs. OC: b = -3.10, 95% CI [-3.87, -2.33], t(153) = -7.96, p < .001
  - IUD vs. OC: b = -0.88, 95% CI [-1.66, -0.11], t(153) = -2.25, p = 0.026
  - T1 vs. T2: b = 0.81, 95% CI [0.30, 1.33], t(153) = 3.13, p = 0.002
- Hydroxyprogesterone
  - NC vs. IUD: b = -0.96, 95% CI [-1.40, -0.52], t(149) = -4.35, p < .001
  - NC vs. OC: b = -2.48, 95% CI [-2.91, -2.05], t(149) = -11.36, p < .001
  - IUD vs. OC: b = -1.52, 95% CI [-1.95, -1.08], t(149) = -6.85, p < .001

- Pregnenolone
  - NC vs. IUD: b = -0.40, 95% CI [-0.78, -0.02], t(152) = -2.10, p = 0.038
  - NC vs. OC: b = -1.13, 95% CI [-1.50, -0.75], t(152) = -5.96, p < .001
  - IUD vs. OC: b = -0.73, 95% CI [-1.10, -0.35], t(152) = -3.82, p < .001
- Allopregnanolone
  - NC vs. IUD: b = -1.25, 95% CI [-1.80, -0.70], t(150) = -4.48, p < .001
  - NC vs. OC: b = -2.18, 95% CI [-2.73, -1.63], t(150) = -7.87, p < .001
  - IUD vs. OC: b = -0.93, 95% CI [-1.48, -0.38], t(150) = -3.32, p = 0.001
- Oestradiol
  - NC vs. IUD: b = -0.10, 95% CI [-0.19, -0.01], t(125) = -2.28, p = 0.024
  - NC vs. OC: b = -0.36, 95% CI [-0.46, -0.25], t(125) = -6.70, p < .001
  - IUD vs. OC: b = -0.25, 95% CI [-0.36, -0.15], t(125) = -4.74, p < .001
- Estrone
  - NC vs. IUD: b = -0.72, 95% CI [-1.18, -0.25], t(126) = -3.07, p = 0.003
  - NC vs. OC: b = -2.01, 95% CI [-2.46, -1.55], t(126) = -8.78, p < .001
  - IUD vs. OC: b = -1.29, 95% CI [-1.74, -0.85], t(126) = -5.75, p < .001
- Estriol
  - not significant
- Corticosterone
  - T1 vs. T2: b = -0.26, 95% CI [-0.49, -0.04], t(150) = -2.29, p = 0.024
- Cortisone
  - NC vs. IUD: b = -0.27, 95% CI [-0.47, -0.07], t(153) = -2.61, p = 0.010
  - NC vs. OC: b = 0.34, 95% CI [0.14, 0.54], t(153) = 3.34, p = 0.001
  - IUD vs. OC: b = 0.61, 95% CI [0.41, 0.81], t(153) = 5.94, p < .001
  - T1 vs. T2: b = 0.21, 95% CI [0.05, 0.38], t(153) = 2.53, p = 0.012
- Cortisol
  - NC vs. IUD: not significant
  - NC vs. OC: b = 0.68, 95% CI [0.50, 0.86], t(150) = 7.35, p < .001
  - IUD vs. OC: b = 0.82, 95% CI [0.64, 1.00], t(150) = 8.92, p < .001
- Levonorgestrel
  - IUD vs. OC: b = 3.56, 95% CI [3.17, 3.94], t(69) = 18.58, p < .001
  - T1 vs. T2: b = 0.35, 95% CI [0.18, 0.53], t(69) = 4.09, p < .001

Supplement Table S3. Steroid hormones

Values indicate means and SD in parentheses.

Hormone (in nmol/L)	IUD (n=27)		OC (n=30)		NC (n=29)		Significant Outcomes*
	T1	T2	T1	T2	T1	T2	
**Endogenous hormones**							
Testosterone	0.48 (0.33)	0.79 (0.70)	0.52 (0.52)	0.89 (0.61)	0.80 (0.74)	0.92 (0.85)	T1 < T2
Dihydrotestosterone	0.30 (0.23)	0.44 (0.45)	0.36 (0.39)	0.54 (0.45)	0.65 (0.71)	0.56 (0.42)	IUD = OC < NC
Progesterone	9.63 (13.24)	17.71 (46.60)	2.05 (4.26)	5.97 (8.35)	111.04 (271.45)	65.59 (115.68)	OC < IUD < NC & T1 < T2
Hydroxyprogesterone[a]	1.63 (1.19)	1.62 (1.81)	0.24 (0.17)	0.41 (0.33)	4.05 (4.35)	3.97 (3.28)	OC < IUD < NC
Pregnenolone	9.60 (6.16)	12.67 (12.55)	6.05 (6.52)	5.50 (4.15)	19.64 (12.01)	14.53 (14.52)	OC < IUD < NC
Allopregnanolone[b]	0.63 (0.70)	0.89 (1.15)	0.18 (0.22)	0.30 (0.35)	4.72 (11.14)	1.65 (2.01)	OC < IUD < NC
Oestradiol[c]	0.33 (0.25)	0.25 (0.20)	0.04 (0.11)	0.04 (0.10)	0.39 (0.23)	0.40 (0.27)	OC < IUD < NC
Estrone	2.90 (2.23)	2.80 (1.97)	2.78 (11.84)	2.06 (7.28)	4.82 (3.29)	5.21 (3.89)	OC < IUD < NC
Estriol[d]	0.01 (0.01)	0.00 (0.00)	0.00 (0.00)	0.00 (0.01)	0.01 (0.01)	0.01 (0.00)	-
Corticosterone	5.69 (3.19)	7.11 (7.47)	11.47 (11.10)	7.32 (5.21)	12.16 (18.26)	6.85 (6.52)	T2 < T1
Cortisone	30.39 (7.41)	42.17 (18.09)	65.09 (46.67)	87.72 (70.98)	48.37 (34.31)	66.17 (76.29)	IUD < NC < OC & T1 < T2

Cortisol	145.88 (45.04)	177.22 (94.99)	363.31 (164.44)	368.57 (157.65)	200.01 (68.51)	203.44 (156.19)	IUD = NC < OC
**Exogenous hormones**							
Ethinyl oestradiol	-	-	0.42 (0.38)	0.34 (0.17)	-	-	-
Cyproterone acetate[e]	-	-	11.64 (-)	-	-	-	-
Chlormadinone acetate[f]	-	-	1.01 (0.25)	2.39 (1.18)	-	-	-
Dienogest[g]	-	-	68.95 (56.54)	107.81 (95.67)	-	-	-
Levonorgestrel[h]	0.22 (0.18)	0.36 (0.48)	8.46 (6.64)	11.00 (6.57)	-	-	IUD < OC, T1 < T2

Note. Deviating sample sizes: [a]n(IUD)=26; [b]n(OC)=28; [c]n(OC)=13; [d]n(IUD)=7, n(OC)=4, n(NC)=19; [e]n(OC)=1; [f]n(OC)=4; [g]n(OC)=8; [h]n(OC)=17. *Statistical values see supplementary results above.

Supplement Table S4. Steroid hormone references values

Serum hormone levels	Lower value (in nmol/L)	Upper value (in nmol/L)
Testosterone[a, 1]	0.07	1.6
Dihydrotestosterone[a, 1]	0.17	1.6
Progesterone[a, 2]	-	≤100
Pregnenolone[b, 2]	2.46	29.67
Allopregnanolone[c, 1]	0.13	3.70
Hydroxyprogesterone[a, 2]	1.0	8.7
Oestradiol[a, 2]	0.176	1.615
Estrone[a, 2]	0.055	0.740
Estriol[a]	No values for non-pregnant women	
Corticosterone[b, 2]	1.88	36.1
Cortisone[b, 2]	34.4	92.1
Cortisol (around 4:00 pm)[a, 1]	83	441

Note. Sources: a: Rifai, 2017 [25]; b: Eisenhofer et al., 2017 [26]; c: Nyberg et al., 2007 [27]. 1: NC women across the menstrual cycle; 2: NC women in the luteal phase.

Repeated measures matter for ANS but not cortisol or subjective stress

Physiological stress (T1 vs. T2):

- Mean HR: b = -2.55, 95% CI [-3.99, -1.11], t(254) = -3.49, p < .001
- Frequency of SCRs: b = -1.53, 95% CI [-2.20, -0.87], t(312) = -4.54, p < .001
- Tonic SC: b = -1.88, 95% CI [-2.29, -1.48], t(311) = -9.08, p < .001
- Global SC: b = -1.94, 95% CI [-2.36, -1.51], t(311) = -9.01, p < .001

Successful Stress Induction

Subjective stress and affect (placebo vs. stress):

- negative affect: b = 0.57, 95% CI [0.41, 0.74], t(310) = 6.89, p < .001
- subjective stress: b = 40.07, 95% CI [33.96, 46.17], t(311) = 12.91, p < .001
- anxiety: b = 12.12, 95% CI [8.67, 15.57], t(311) = 6.91, p < .001

Cortisol response (placebo vs. stress):

- AUCi: b = 103.23, 95% CI [61.28, 145.18], t(293) = 4.84, p < .001

Physiological stress (placebo vs. stress):

- HR: b = 6.27, 95% CI [4.11, 8.43], t(254) = 5.72, p < .001
- SCR frequency: b = 5.18, 95% CI [4.06, 6.31], t(312) = 9.10, p < .001
- tonic SC: b = 1.79, 95% CI [1.11, 2.48], t(311) = 5.15, p < .001
- global SC: b = 1.98, 95% CI [1.27, 2.69], t(311) = 5.50, p < .001

Subjective stress altered in IUDs, cortisol in OCs

Subjective stress and affect:

- NC vs. IUD
    - Positive affect: b = -0.37, 95% CI [-0.65, -0.10], t(311) = -2.65, p = 0.008
    - Negative affect: b = 0.43, 95% CI [0.20, 0.66], t(310) = 3.66, p < .001
    - Subjective stress: b = 13.28, 95% CI [4.70, 21.85], t(311) = 3.05, p = 0.003
    - Anxiety: b = 7.40, 95% CI [2.49, 12.30], t(311) = 2.97, p = 0.003
- IUD vs. OC
    - Negative affect: b = -0.31, 95% CI [-0.54, -0.08], t(310) = -2.65, p = 0.009

Cortisol response:

- NC vs. OC
    - AUCi: b = -80.14, 95% CI [-137.93, -22.35], t(293) = -2.73, p = 0.007
- OC vs. IUD
    - AUCi: b = -62.54, 95% CI [-119.91, -5.17], t(293) = -2.15, p = 0.033

Physiological stress:

- NC vs. OC
    - Mean HR: b = 6.52, 95% CI [1.15, 11.88], t(254) = 2.39, p = 0.018

Daily Diary shows lower negative affect and stress in OCs

Detailed EMA values can be found in supplement Table S5 below. Significant statistical outcomes:
IUD vs. OC:

- Negative affect: b = -1.20, 95% CI [-2.21, -0.19], t(104) = -2.35, p = 0.021
- Negative emotions: b = -42.08, 95% CI [-78.33, -5.83], t(128) = -2.30, p = 0.023
- Acute work stress: b = -64.34, 95% CI [-118.24, -10.43], t(130) = -2.36, p = 0.020
- Anger: b = -1.49, 95% CI [-2.78, -0.20], t(127) = -2.28, p = 0.024
- Sadness: b = -0.87, 95% CI [-1.61, -0.14], t(124) = -2.36, p = 0.020

NC vs. OC:

- Negative emotions: b = -41.34, 95% CI [-76.83, -5.86], t(128) = -2.31, p = 0.023
- Acute socio-emotional stress: b = -51.12, 95% CI [-94.58, -7.66], t(130) = -2.33, p = 0.021

Supplement Table S5. 7-day-diary variables and statistics.

Mean values for the areas under the curve for each 7-day-diary variable.

AUCg for 7-day EMA, mean (SD)	IUD (n=27)		OC (n=30)		NC (n=29)		Significant outcomes*
	T1	T2	T1	T2	T1	T2	
Positive Affect	16.31 (3.03)	17.62 (3.08)	16.45 (4.05)	16.19 (4.55)	17.49 (3.27)	17.62 (4.00)	-
Negative Affect	8.91 (1.84)	9.12 (2.45)	7.72 (1.16)	7.95 (1.40)	8.50 (1.87)	8.51 (2.12)	OC < IUD
State Anxiety	236.72 (48.64)	223.97 (43.58)	217.04 (44.02)	226.33 (43.10)	235.53 (42.98)	226.78 (40.00)	-
I feel good	212.27 (90.99)	190.01 (70.65)	196.83 (90.62)	212.02 (79.07)	192.65 (67.86)	214.83 (86.17)	-
I feel stressed	220.79 (91.15)	236.10 (82.75)	212.76 (102.42)	230.04 (112.85)	224.39 (96.91)	232.16 (91.99)	-
Positive emotions	191.14 (80.25)	181.73 (73.43)	190.90 (84.26)	219.34 (81.73)	187.01 (73.16)	208.47 (98.26)	-
Negative emotions	172.09 (80.86)	163.61 (83.61)	137.87 (86.31)	117.76 (77.67)	160.08 (71.88)	180.31 (75.85)	OC < IUD = NC
Acute socio-emotional stress	156.44 (106.91)	162.50 (89.49)	117.98 (110.67)	127.35 (73.76)	186.18 (93.68)	164.26 (79.24)	OC < NC
Acute work stress	194.44 (129.64)	149.53 (111.23)	110.72 (97.53)	120.99 (119.13)	129.60 (95.15)	137.24 (92.53)	OC < IUD
Acute physical/medical stress	91.15 (64.15)	104.20 (120.72)	68.35 (79.25)	58.49 (65.01)	96.02 (73.71)	71.80 (89.35)	-
Anger	10.04 (2.77)	9.74 (3.46)	8.79 (2.95)	8.18 (2.60)	8.75 (2.55)	8.90 (2.21)	OC < IUD
Happiness	19.72 (3.12)	20.56 (2.66)	20.11 (3.93)	20.07 (3.91)	21.22 (3.80)	20.24 (4.61)	-
Sadness	7.34 (1.92)	7.68 (1.73)	6.76 (1.38)	6.47 (1.14)	7.15 (1.61)	6.95 (1.43)	OC < IUD
Fear	7.48 (2.55)	7.37 (2.34)	6.50 (1.57)	6.72 (0.94)	7.40 (2.04)	6.67 (0.90)	-

Note. *Statistical values see supplementary results.

Sex hormones affect stress response

Association of progesterone with tonic and global SC

- For NC-women:
  - tonic SC: b = -0.52, 95% CI [-0.90, -0.15], t(36) = 2.71, p_{FDR} = 0.031
  - global mean SC: b = -0.54, 95% CI [-0.94, -0.15], t(36) = 2.67, p_{FDR} = 0.034
- For IUD-users;
  - tonic SC: b = -0.52, 95% CI [-0.98, -0.05], t(39) = 2.18, p_{FDR} = 0.111
  - global mean SC: b = -0.53, 95% CI [-1.01, -0.05], t(39) = 2.15, p_{FDR} = 0.124
- For OC-users
  - tonic SC: b = -0.58, 95% CI [-1.07, -0.10], t(42) = 2.34, p_{FDR} = 0.121
  - global mean SC: b = -0.60, 95% CI [-1.11, -0.09], t(42) = 2.31, p_{FDR} = 0.131

Association of testosterone with acute socio-emotional stress (EMA)

- IUD-users: b = -28.07, 95% CI [-69.78, 13.64], t(36) = 1.30, p_{uncorr}=0.202
- OC-users: b = -20.79, 95% CI [-65.79, 25.24], t(39) = 0.91, p_{uncorr}=0.37
- NC-women: b = 37.34, 95% CI [9.68, 65.69], t(25) = 2.61, p_{FDR}=0.0456

Association of oestradiol with negative affect (EMA)

- IUD-users: b = -3.39, 95% CI [-5.36, -1.29], t(25) = 3.38, p_{FDR} = 0.01
- OC-users: b = -0.69, 95% CI [-7.02, 5.94], t(14) = 0.21, p_{uncorr} = 0.839
- NC-women: b = 0.79, 95% CI [-1.43, 2.99], t(37) = 0.70, p_{uncorr} = 0.491

Supplementary Tables (referenced in manuscript)

Supplement Table S6. Number of women for each task presentation order

	IUD	OC	NC	statistics
**Timepoint T1**				
**placebo 1st – stress 2nd**	13	17	14	
**stress 1st – placebo 2nd**	14	13	15	$\chi^2(2)=0.559$, $p = .756$
**Timepoint T2**				
**placebo 1st – stress 2nd**	14	13	16	
**stress 1st – placebo 2nd**	13	17	13	$\chi^2(2)=0.881$, $p=.644$

Supplement Table S7. 7-day-diary variables

Original German version	English Translation
• Ich fühle mich: ruhig – unruhig	• I feel: calm – restless
• Ich fühle mich gut: überhaupt nicht – extrem	• I feel good: not at all - extremely
• Ich bin unzufrieden mit mir: überhaupt nicht – extrem	• I am dissatisfied with myself: not at all - extremely
• Der heutige Tag war: angenehm – unangenehm	• Today was: pleasant - unpleasant
• Der heutige Tag war: positiv – negativ	• Today was: positive - negative
• Wie sehr fühlten Sie sich heute gestresst: überhaupt nicht – extrem	• How stressed did you feel today: not at all - extremely stressed
• PANAS[22]	• PANAS[22]
• ESR[23]	• ESR[23]
• STAI-S[12]	• STAI-S[12]
• Ich hatte heute positive Erlebnisse	• I had positive experiences today
○ Positive soziale Erlebnisse: überhaupt nicht – extrem	○ Positive social experiences: not at all - extremely
○ Glücksmomente: überhaupt nicht – extrem	○ Moments of happiness: not at all - extremely
○ Positive Emotionen (Stolz, Dankbarkeit, Freude, Glücksempfindung, Seelenruhe, Zufriedenheit, Flow, etc.): überhaupt nicht – extrem	○ Positive emotions (pride, gratitude, joy, feeling of happiness, peace of mind, satisfaction, flow, etc.): not at all - extreme
• Ich hatte heute negative Erlebnisse	• I had negative experiences today
○ Negative soziale Erlebnisse : überhaupt nicht – extrem	○ Negative social experiences: not at all - extremely
○ Negative Emotionen (Ärger, Wut, Trauer, Angst, Scham, etc.): überhaupt nicht – extrem	○ Negative emotions (anger, rage, sadness, fear, shame, etc.): not at all - extremely
• Heute sind folgende Belastungen aufgetreten:	• The following stressors have occurred today:

○ Alltagsbelastung (täglich wiederkehrende kleine Ärgernisse/Belastungen im Alltag): überhaupt nicht – extrem	○ Everyday stress (daily recurring minor annoyances/stress in everyday life): not at all - extreme
○ Akute Belastung (sozial, emotional/mental/psychisch): überhaupt nicht – extrem	○ Acute stress (social, emotional/mental/psychological): not at all - extreme
○ Akute Arbeitsbelastung: überhaupt nicht – extrem	○ Acute workload: not at all - extreme
○ Akute körperlich-medizinische Belastung/Schmerzen: überhaupt nicht – extrem	○ Acute physical/medical stress/pain: not at all - extreme
○ Chronische Belastungen (sozial, emotional/mental/psychisch): überhaupt nicht – extrem	○ Chronic stress (social, emotional/mental/psychological): not at all - extreme
○ Chronische Arbeitsbelastung: überhaupt nicht – extrem	○ Chronic workload: not at all - extreme
○ Chronische körperlich-medizinische Belastungen/Schmerzen: überhaupt nicht - extrem	○ Chronic physical/medical stress/pain: not at all - extreme
○ Andere chronisch-körperlilche Belastungen (Sport, etc.): überhaupt nicht – extrem	○ Other chronic physical stress (sport, etc.): not at all - extreme
• Offene Frage für Kommentare	• Open question for comments

Supplementary Figures (referenced in manuscript)

Supplement Figure S1. Cortisol AUCi

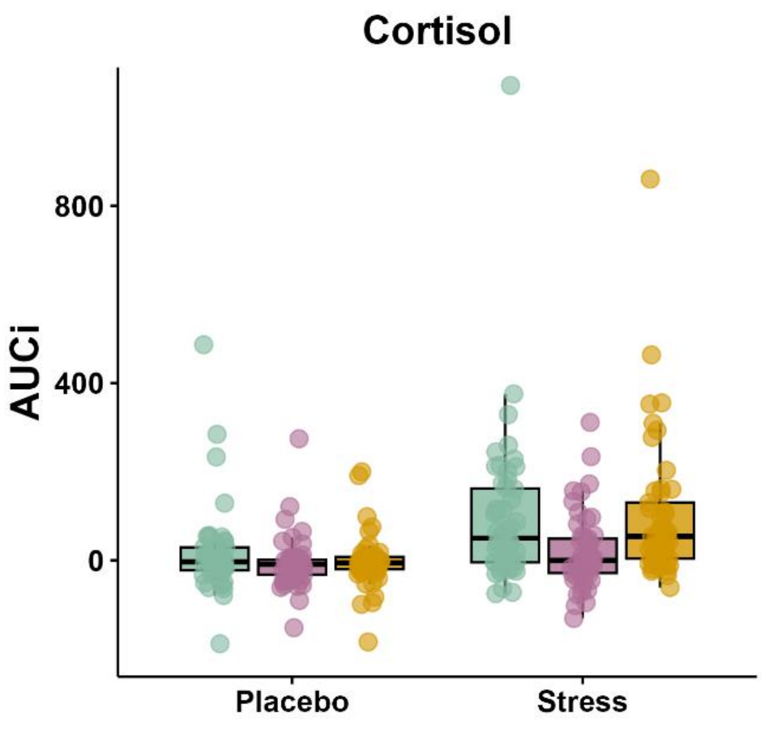

Figure S1. Cortisol AUCi for IUD-users (in green), OC-users (pink) and NC women (orange).

Supplement Figure S2. Cortisone

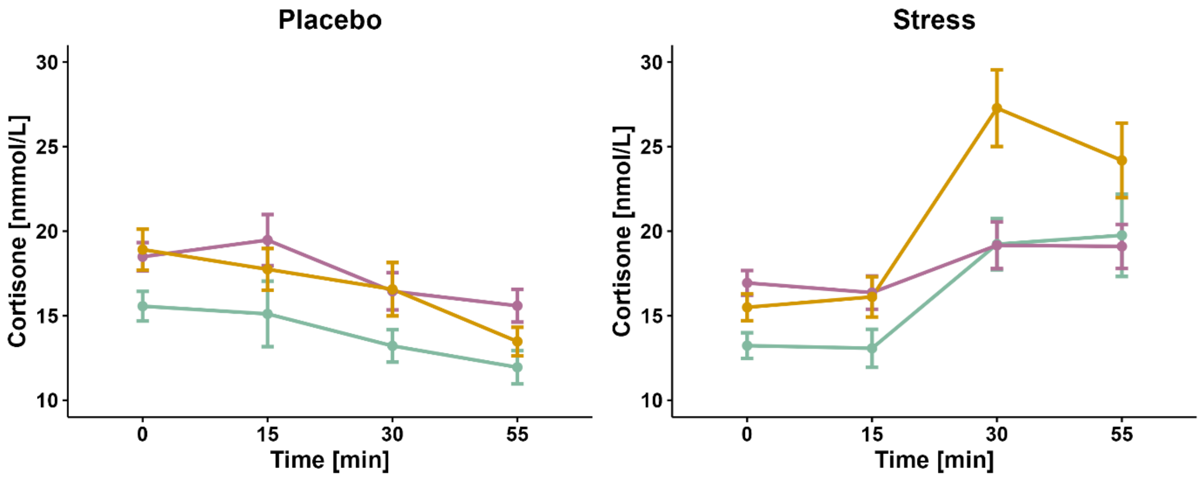

Figure S2. Cortisone reactivity during placebo vs. stress condition across IUD-users (green), OC-users (pink) and NC women (orange). Stress/Placebo onset at time 0, until time 15, dots and bars indicate mean and standard error. Cortisone AUCi is significantly higher in stress vs. placebo (b=299.69, 95%CI [178.62, 420.77], t(305)=4.87, p<0 .001), and OC-users have significantly lower AUCi compared with NC-women in stress (b=-97.02, 95%CI [-270.73, 76.69], t(305)=-1.10, p=0.273).

Supplement Figure S3. Cortisol-to-cortisone ratio

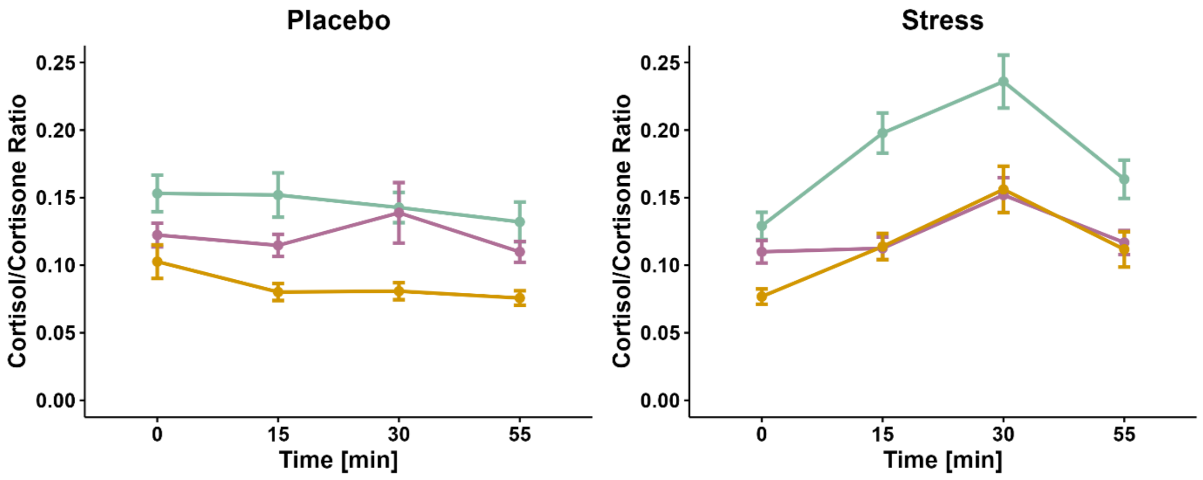

Figure S3. Ratio of cortisol-to-cortisone during placebo vs. stress condition across IUD-users (green), OC-users (pink) and NC women (orange). Stress/Placebo onset at time 0 until time 15, dots and bars indicate mean and standard error. Cortisol-to-cortisone ratio AUCi is significantly higher in stress than placebo (b=2.49, 95%CI [1.29, 3.68], t(291)=4.09, p<0.001) and during stress, AUCi for OC-users was significantly lower than for NC-women (Nc vs. OC: b=-2.16, 95%CI [-3.80, -0.51], t(291)=2.58, p=0.010) and IUD-users (IUD vs. OC: b=-2.60, 95%CI [-4.25, -0.95], t(291)=3.11, p=0.002).

References

1 Schmalenberger KM, Tauseef HA, Barone JC, Owens SA, Lieberman L, Jarczok MN, *et al.* How to study the menstrual cycle: Practical tools and recommendations. *Psychoneuroendocrinology* 2021; **123**: 104895.

2 Hampson E. A brief guide to the menstrual cycle and oral contraceptive use for researchers in behavioral endocrinology. *Hormones and Behavior* 2020; **119**: 104655.

3 Gloe LM, Russman Block S, Klump KL, Beltz AM, Moser JS. Determining menstrual cycle phase: An empirical examination of methodologies and recommendations for improvement in behavioral and brain sciences. *Horm Behav* 2023; **155**: 105421.

4 Juster R-P, Hatzenbuehler ML, Mendrek A, Pfaus JG, Smith NG, Johnson PJ, *et al.* Sexual orientation modulates endocrine stress reactivity. *Biol Psychiatry* 2015; **77**: 668–76.

5 Wittchen H, Wunderlich U, Gruschwitz S, Zaudig M. Skid i. Strukturiertes klinisches interview für dsm-iv. Achse i: psychische störungen. Interviewheft und beurteilungsheft. Eine deutschsprachige, erweiterte. 1997. (https://pure.mpg.de/pubman/faces/ViewItemOverviewPage.jsp?itemId=item_1646480).

6 Steiner M, Macdougall M, Brown E. The premenstrual symptoms screening tool (PSST) for clinicians. *Arch Womens Ment Health* 2003; **6**: 203–9.

7 Lac G, Chamoux A. Biological and psychological responses to two rapid shiftwork schedules. *Ergonomics* 2004; **47**: 1339–49.

8 Casto KV, Edwards DA. Testosterone, cortisol, and human competition. *Hormones and Behavior* 2016; **82**: 21–37.

9 Gobrecht-Keller U. Gestagengabe in der Menopause: Was sind Unterschiede, Vorteile und Nachteile der einzelnen Präparate?: Eine aktuelle Übersicht. *J Gynäkol Endokrinol CH* 2021; **24**: 58–68.

10 Smeets T, Cornelisse S, Quaedflieg CWEM, Meyer T, Jelicic M, Merckelbach H. Introducing the Maastricht Acute Stress Test (MAST): a quick and non-invasive approach to elicit robust autonomic and glucocorticoid stress responses. *Psychoneuroendocrinology* 2012; **37**: 1998–2008.

11 Kühner C, Bürger C, Keller F, Hautzinger M. Reliabilität und Validität des revidierten Beck-Depressionsinventars (BDI-II). *Nervenarzt* 2007; **78**: 651–6.

12 Laux L. Das State-Trait-Angstinventar (STAI) : theoretische Grundlagen und Handanweisung. 1981. (https://fis.uni-bamberg.de/handle/uniba/26756).

13 Wingenfeld K, Spitzer C, Mensebach C, Grabe HJ, Hill A, Gast U, *et al.* [The German Version of the Childhood Trauma Questionnaire (CTQ):Preliminary Psychometric Properties.]. *Psychother Psychosom Med Psychol* 2010; **60**: e13.

14 Ungerer O, Deter H-C, Fikentscher E, Konzag TA. [Improved diagnostics of trauma-related disease through the application of the Life-Stressor Checklist]. *Psychother Psychosom Med Psychol* 2010; **60**: 434–41.

15 *WHOQOL - 100 und WHOQOL - BREF Handbuch für die deutschsprachige Version der WHO-Instrumente zur Erfassung von Lebensqualität*. Hogrefe, 2000 (https://d-nb.info/959636390/04).

16 von Collani G, Herzberg PY. Eine revidierte Fassung der deutschsprachigen Skala zum Selbstwertgefühl von Rosenberg. *Zeitschrift für Differentielle und Diagnostische Psychologie* 2003; **24**: 3–7.

17 Schulz P, Schlotz W, Becker P. Trierer Inventar zum Chronischen Stress (TICS) [Trier Inventory for Chronic Stress (TICS)]. Hogrefe, 2004 (https://eprints.soton.ac.uk/50017/).

18 Reis D, Lehr D, Heber E, Ebert DD. The German Version of the Perceived Stress Scale (PSS-10): Evaluation of Dimensionality, Validity, and Measurement Invariance With Exploratory and Confirmatory Bifactor Modeling. *Assessment* 2019; **26**: 1246–59.

19 Endler NS, Parker JDA. Assessment of multidimensional coping: Task, emotion, and avoidance strategies. *Psychological Assessment* 1994; **6**: 50–60.

20 Abler B, Kessler H. Emotion Regulation Questionnaire – Eine deutschsprachige Fassung des ERQ von Gross und John. *Diagnostica* 2009; **55**: 144–52.

21 Rosen CB J Heiman, S Leiblum, C Meston, R Shabsigh, D Ferguson, R D'Agostino ,R. The Female Sexual Function Index (FSFI): A Multidimensional Self-Report Instrument for the Assessment of Female Sexual Function. *Journal of Sex & Marital Therapy* 2000; **26**: 191–208.

22 Krohne HW, Egloff B, Kohlmann C-W, Tausch A. Untersuchungen mit einer deutschen version der" positive and negative affect schedule"(PANAS). *researchgate.net* 2014. doi:10.1037/t49650-000.

23 Schneider F, Gur RC, Gur RE, Muenz LR. Standardized mood induction with happy and sad facial expressions. *Psychiatry Research* 1994; **51**: 19–31.

24 Benedek M, Kaernbach C. A continuous measure of phasic electrodermal activity. *Journal of Neuroscience Methods* 2010; **190**: 80–91.

25 Rifai N. *Tietz Textbook of Clinical Chemistry and Molecular Diagnostics - E-Book: Tietz Textbook of Clinical Chemistry and Molecular Diagnostics - E-Book*. Elsevier Health Sciences, 2017.

26 Eisenhofer G, Peitzsch M, Kaden D, Langton K, Pamporaki C, Masjkur J, *et al.* Reference intervals for plasma concentrations of adrenal steroids measured by LC-MS/MS: Impact of gender, age, oral contraceptives, body mass index and blood pressure status. *Clinica Chimica Acta* 2017; **470**: 115–24.

27 Nyberg S, Bäckström T, Zingmark E, Purdy RH, Poromaa IS. Allopregnanolone decrease with symptom improvement during placebo and gonadotropin-releasing hormone agonist treatment in women with severe premenstrual syndrome. *Gynecological Endocrinology* 2007; **23**: 257–66.
